# Cardiovascular correlates of sleep apnea phenotypes: Results from the Hispanic Community Health Study/Study of Latinos (HCHS/SOL)

**DOI:** 10.1371/journal.pone.0265151

**Published:** 2022-04-04

**Authors:** Benson Wu, Wassim Tarraf, Douglas M. Wallace, Ariana M. Stickel, Neil Schneiderman, Susan Redline, Sanjay R. Patel, Linda C. Gallo, Yasmin Mossavar-Rahmani, Martha L. Daviglus, Phyllis C. Zee, Gregory A. Talavera, Daniela Sotres-Alvarez, Hector M. González, Alberto Ramos

**Affiliations:** 1 Department of Neurosciences and Shiley-Marcos Alzheimer’s Disease Research Center, University of California San Diego School of Medicine, San Diego, California, United States of America; 2 Department of Healthcare Sciences and Institute of Gerontology, Wayne State University, Detroit, Michigan, United States of America; 3 Department of Neurology, University of Miami Miller School of Medicine, Miami, Florida, United States of America; 4 Department of Psychology, University of Miami, Miami, Florida, United States of America; 5 Department of Medicine, Beth Israel Deaconess Medical Center, Harvard Medical School, Boston, Massachusetts, United States of America; 6 Department of Medicine, University of Pittsburgh Medical Center, Pittsburgh, Pennsylvania, United States of America; 7 Department of Psychology and South Bay Latino Research Center, San Diego State University, San Diego, California, United States of America; 8 Department of Epidemiology and Population Health, Albert Einstein College of Medicine, Bronx, New York, United States of America; 9 Institute for Minority Health Research, University of Illinois at Chicago College of Medicine, Chicago, Illinois, United States of America; 10 Department of Neurology, Northwestern University Feinberg School of Medicine, Chicago, Illinois, United States of America; 11 Graduate School of Public Health, San Diego State University, San Diego, California, United States of America; 12 Department of Biostatistics, University of North Carolina Gillings School of Global Public Health, Chapel Hill, North Carolina, United States of America; Sapienza University of Rome, ITALY

## Abstract

**Background:**

Identifying Obstructive Sleep Apnea (OSA) phenotypes among middle-aged and older Hispanics/Latinos can facilitate personalized care, better inform treatment decisions, and could lead to improved clinical outcomes.

**Methods:**

We focused on middle-aged and older adults (ages ≥45–74 years at baseline) with an apnea-hypopnea index (AHI) ≥5 from the HCHS/SOL (2008–2011) (unweighted n = 3,545). We used latent class analyses (LCA) to identify empirical and clinically meaningful OSA phenotypes. Sleep variables included AHI, percent sleep time SpO2<90%, Epworth Sleepiness Scale (ESS), Women’s Health Initiative Insomnia Rating Scale (WHIIRS) score, self-reported average sleep duration, restless legs symptoms, napping frequency, and self-reported sleep quality. We used survey logistic and Poisson regression to test the associations between our OSA phenotypes and prevalent and incident cardiovascular measures (cardiovascular disease, heart failure, Stroke/TIA, hypertension, diabetes, and the Framingham Cardiovascular Risk Score).

**Results:**

Average AHI, ESS, WHIIRS, and sleep duration were 18.1±19.5, 6.3±6.1, 7.4±6.6, and 7.8±1.7 hours, respectively, and 2.9% had zero percent time SpO2 <90%. We identified a three-class solution that clustered individuals into (1) *insomnia OSA* (44.3%), (2) *asymptomatic mild OSA*, (36.2%) and (3) *symptomatic OSA* (19.5%). Elevated WHIIRS and AHI scores primarily drove classification into groups one and three, respectively. In covariate adjusted models, OSA phenotypes were differentially associated with prevalence (baseline and seven years later) and incidence of cardiovascular measures.

**Conclusions:**

OSA subtypes in diverse U.S. Hispanic/Latino adults have different cardiovascular complications. More targeted research, that takes these variations into account, could help ameliorate Hispanic/Latino sleep and cardiovascular health disparities.

## Introduction

US health disparities are well recognized, broad, persistent, and include sleep [1–3]. Sleep disorders, such as obstructive sleep apnea (OSA) adversely affects a high proportion of minorities and is expected to increase over the next few decades as the older US population grows and diversifies. The healthcare cost of OSA exceeded $149.6 billion in 2015 [2]. Understanding the burden of sleep disorders is a critical public health need. For example, OSA [2] has functional and health implications that include increased mortality, motor vehicle accidents, daytime sleepiness, metabolic disease and stroke [4–8]. A recent scientific statement from the American Heart Association highlights the importance of diagnosing and treating OSA in cardiovascular practices given its high prevalence among patients with cardiovascular risk (e.g. hypertension), disease (e.g. heart failure), and stroke [9]. Though the mechanisms explaining the associations between OSA and cardiovascular disease are not completely understood, several intermediate mechanisms such as sustained sympathetic activation, change in intrathoracic pressure, and oxidative stress have been proposed [10–12]. Our published data from the Hispanic Community Health Study/Study of Latinos (HCHS/SOL), the largest study of Hispanic/Latinos in the U.S., showed an OSA prevalence of 34% in Hispanic/Latino men and of 18% in Hispanic/Latino women. In addition, OSA had strong associations with obesity, hypertension and diabetes mellitus, leading comorbidities in Hispanics/Latinos, yet an estimated 93% of Hispanic/Latino women and 82% of men with OSA remain untreated [13]. To date, the treatment of OSA has not consistently reduced vascular risk in clinical trials nor consistently improved other important health outcomes [14]. There is also a paucity of minorities in OSA clinical trials; therefore, identifying OSA phenotypes and their links to cardiovascular outcomes can lead to treatment studies for personalized care and better treatment strategies. The apnea-hypopnea index (AHI) is the main diagnostic and treatment metric for OSA [15]. However, this metric does not consider the heterogeneity of OSA mechanisms, symptoms, clinical presentations, and outcomes [15]. OSA reduces or ceases the air flow in upper airways, which leads to activation of an inflammatory cascade. Ongoing research has found that novel biomarkers, such as tumor necrosis factors, inflammatory cytokines, lipid peroxidation, and cell-free DNA, increase in OSA patients [16]. Therefore, focusing on the AHI only could lead to (1) suboptimal understanding of the variations in OSA pathology and risk factors; (2) a lack of appropriate clinical understanding of variability in OSA symptom presentations; (3) inability to provide specific sleep disease treatment; and (4) limited impact on modifying sleep risk factors on distal older age disease outcomes such as stroke and dementia.

The majority of studies based on largely white samples define OSA *a priori* such that an individual has an AHI≥5 with common symptoms (e.g. daytime sleepiness) [17]. However, daytime sleepiness was a less common presentation, in studies using cluster analysis to define OSA phenotypes [18]. Existing studies have pointed to other sleep metrics, clinical symptoms, and molecular markers that could be better predictors of comorbid outcomes in OSA [19]. A data-driven approach can provide new understanding of sleep symptoms as well as demographic (e.g., ethnicity and race), clinical, and physiological data, particularly related to cardiovascular risk and disease, that define heterogeneous OSA phenotypes [19–23]. Doing so in diverse and understudied groups is critical for developing better-tailored prevention strategies, clinical interventions, and therapeutics.

Recent studies suggest that OSA symptom clusters vary by demographic characteristics [24]. Current evidence, however, remains limited by lack of validation and reproducibility in large non-clinical samples, data with sufficient female representation and diverse and racial/ethnical populations. We plan to fill this gap by defining OSA phenotypes using clustering techniques on data from a large cohort of community dwelling middle-aged and older diverse Hispanics/Latinos, the HCHS/SOL. We aim to determine: (1) the distinct symptom profiles of middle-aged and older Hispanic/Latinos; (2) whether these phenotypes differ by sociodemographic and cardiovascular features; and (3) examine associations between these phenotypes and prevalent and distal cardiovascular outcomes. We hypothesize that 3 to 4 phenotypes will capture the heterogeneities of OSA in HCHS/SOL [24, 25]. We expect that the OSA phenotypes will differ substantially on sociodemographic, health behaviors. Given that older age is a known risk factor for both cardiovascular disease and OSA, we focus on middle-aged and older adults, 50-years and older. We expect to uncover varying profiles in the associations between OSA phenotypes and the prevalence and incidence of cardiovascular risk factors and disease [26, 27].

## Methods

### Data

HCHS/SOL (2008–2011) is a multisite, prospective cohort study of 16,415 community-dwelling Hispanic/Latino adults (18-74-years old) from multiple background groups. The sampling scheme was designed to produce representative estimates of diverse Hispanic/Latinos in the target areas. Data were collected from field centers in four U.S. cities with diverse Hispanic/Latino populations (Bronx, NY; Chicago, IL; Miami, FL; and San Diego, CA). Each field center recruited about 4,000 eligible, self-identified Hispanic/Latino adults. Detailed HCHS/SOL rationale and sampling methods have been published previously [28, 29]. All participants consented to inclusion in the study, and the HCHS/SOL was approved by the IRB of all participating institutions. IRB #20131007 was also approved for this study by University of Miami IRB.

### Analytic subpopulation

Individuals’ ages 45-years and older are at increased risk for cardiovascular disease and cognitive impairment. Therefore, we restricted the analytic sample to participants 45–74 years of age at Visit 1 (V1; 2008–2011; n = 9,714) for the OSA phenotype derivation analysis. Details of the Visit 1 HCHS/SOL study have been published and cited extensively in literature [28, 29]. We further restricted the analytical sample to only include individuals with OSA based on an AHI≥5, excluding 1,112 individuals without AHI data and 5,057 individuals with AHI<5; individuals with AHI <5 were treated as a control group in the subsequent analysis explained below. The analytic sample size for the OSA phenotype derivation analysis was 3,545.

To examine associations between the derived OSA phenotypes with prevalent and incident cardiovascular risk factors and disease, we focused on individuals from the Study of Latinos-Investigation of Neurocognitive Aging (SOL-INCA; 2016–2018), an ancillary study to the HCHS/SOL, who were 50-years and older at a follow-up visit occurring (on average) 7-years later (n = 6,377). Details on the SOL-INCA aims and design are published elsewhere [30]. Of the 6,377 individuals in SOL-INCA, there were n = 22 participants under the age of 45 and n = 602 participants did not participate in the baseline sleep module and had no AHI data, and as such were excluded from analyses. Additionally for the current study, we excluded n = 104 participants who identified as other/more than one Latino background, and n = 61 participants with missing values on any of the covariates of interest. The analytic sample size for the incident cardiovascular disease analysis was 5,588. A diagram containing the inclusion and exclusion criteria is shown in S1 Fig.

#### Sleep measures at visit 1

HCHS/SOL’s sleep questionnaire was adapted from the Sleep Heart Health Study Sleep Habits Questionnaire that evaluates weekday and weekend bedtime and wake time, napping behaviors, as well as related OSA symptoms such as snoring and witnessed apneas [31]. The following questions were used to determine sleep duration in our sample population: *What time do you usually go to bed*? and *What time do you usually wake up*? Average sleep duration was computed as the weighted average of weekday and weekend sleep (5/7 weekday + 2/7 weekend). The Epworth Sleepiness Scale (ESS), assesses the likelihood of falling asleep in eight common situations, having a total of 24 points. Insomnia questions were adapted from the Women’s Health Initiative Insomnia Rating Scale (WHIIRS), which has a total of 20 points derived from five items scored from 0–4 each. The five items assessed sleep latency, sleep maintenance insomnia, early morning awakening, and overall sleep quality. Self-reported sleep quality was defined as ‘Very sound or restful, Sound or restful, Average quality, Restless, Very restless’ based on response to a probe gauging “Overall [what is your] …typical night’s sleep during the past 4 weeks?” A binary measure of restless legs was constructed based on participants’ affirmative responses to all of the following questions: 1) Do you experience a desire to move your legs because of discomfort or disagreeable sensations in your legs, 2) Do you sometimes feel the need to move to relieve the discomfort, for example by walking, or to relieve the discomfort by rubbing your legs, 3) Are these symptoms worse when you are at rest, with at least temporary relief by activity, and 4) Are these symptoms worse later in the day or at night? Information about weekly napping frequency was self-reported by participants based on the following probe: During a usual week, how many times do you nap for 5 minutes or *more*? All questionaires were administered in either English or Spanish, based on participant’s preference.

### Obstructive sleep apnea

OSA data were collected using the ARES Unicorder 5.2; B-Alert (Carlsbad, CA) [32]. Sleep records were scored at the HCHS/SOL Sleep Reading Center. Respiratory events were identified as a 50% or greater reduction in airflow lasting at least 10 seconds with desaturations at least 3%. The apnea-hypopnea index (AHI) was calculated as the number of events divided by estimated sleep time, using methods described previously [13, 33]. The AHI was modelled as a continuous variable. The American Academy of Sleep Medicine defines OSA types as the following: Mild OSA (AHI of 5–15), Moderate OSA (AHI of 15–30), and Severe OSA, Severe OSA (AHI of more than 30) [34]. Additionally, hemoglobin oxygen saturation (SpO_2_) [13] was assessed using a binary variable to separate individuals with any SpO_2_ time <90% from those without (0 = No, 1 = Yes).

### Baseline demographic, socioeconomic, and health characteristics

We characterize the OSA phenotypes relative to the following demographic and socioeconomic characteristics: age, sex, Hispanic/Latino background (Central American, Cuban, Dominican, Mexican, Puerto Rican, and South American), education (less than high school, high school or equivalent, and greater than high school), and yearly household income (<$30,000, ≥$30,000, and not reported). We also examined measures of occupation, classifying both the occupation held for the longest time and current employment status as one of the following: non-skilled worker, service worker, skilled worker, professional/technical/other office worker, other, retired & not employed, and not retired & not employed. Lastly, we assessed level of acculturation using an adapted version of the Short Acculturation Scale for Hispanics (SASH): (1) language (1 = Only Spanish to 5 = Only English) and (2) social acculturation (1 = All Hispanic/Latino to 5 = All non-Hispanic/non-Latino), both coded so that higher values indicate greater acculturation. Detailed discussion of these subscales are provided elsewhere [35]. Additionally, we examined the distributions of the following cardiovascular health biomarkers across the generated phenotypes: HDL (high-density lipoprotein) cholesterol (mg/dL), total cholesterol (mg/dL), and triglycerides (mg/dL), and BMI (body mass index; kg/m^2^). We also examined smoking status (never, former, current) and alcohol consumption (does not drink alcohol or drinks alcohol).

### Cardiovascular risks and disease

Cardiovascular risks and disease measures were examined at both Visit 1 and Visit 2 (V2). Cardiovascular disease (CVD) was defined by the Framingham Study criterion as a binary (Yes/No) composite if individuals reported any of the following: myocardial infarction, coronary insufficiency, angina, ischemic stroke hemorrhagic stroke, transient ischemic attack, peripheral artery disease, heart disease, and other heart problems [36]. Heart failure and stroke/TIA were based on a self-report (no/yes). Type 2 diabetes was operationalized as no if individuals had normal glucose regulation or impaired glucose tolerance and yes if individuals were diabetic per ADA criteria. Hypertension was used as a binary indicator (no/yes) in line with criteria set by the National Health and Nutritional Examination Survey (NHANES). Lastly, we examined a trichotomous measure of cardiovascular risk based on the 10-year Framingham Risk Score (FRS), using standard thresholds for low (FRS<0.1), moderate (0.1≤FRS<0.2), and high risk (FRS≥0.2). The FRS estimates the probability of having a major cardiovascular event within 10 years. Details of the derivation of the cardiovascular FRS are published elsewhere [37].

### Statistical analysis

First, we used latent class analyses (LCA) to estimate the OSA phenotypes in the target population. Clustering techniques including the use of LCA are data-driven methods used to identify phenotypes of individuals with wide ranging and potentially interacting clinical characteristics [21, 25, 38]. LCA is a mixture model used to isolate groups of individuals sharing unique data patterns and characteristics. LCA produces probabilistic classifications of individuals into groups that could be assessed using established model fit statistics [38]. These smaller groups with more homogeneous symptoms presentations are referred to as phenotypes. A few studies have recently used LCAs to identify subpopulations of patients with OSA and attempted to link these extracted phenotypes to distal clinical outcomes [39–42].

**Fig 1:**
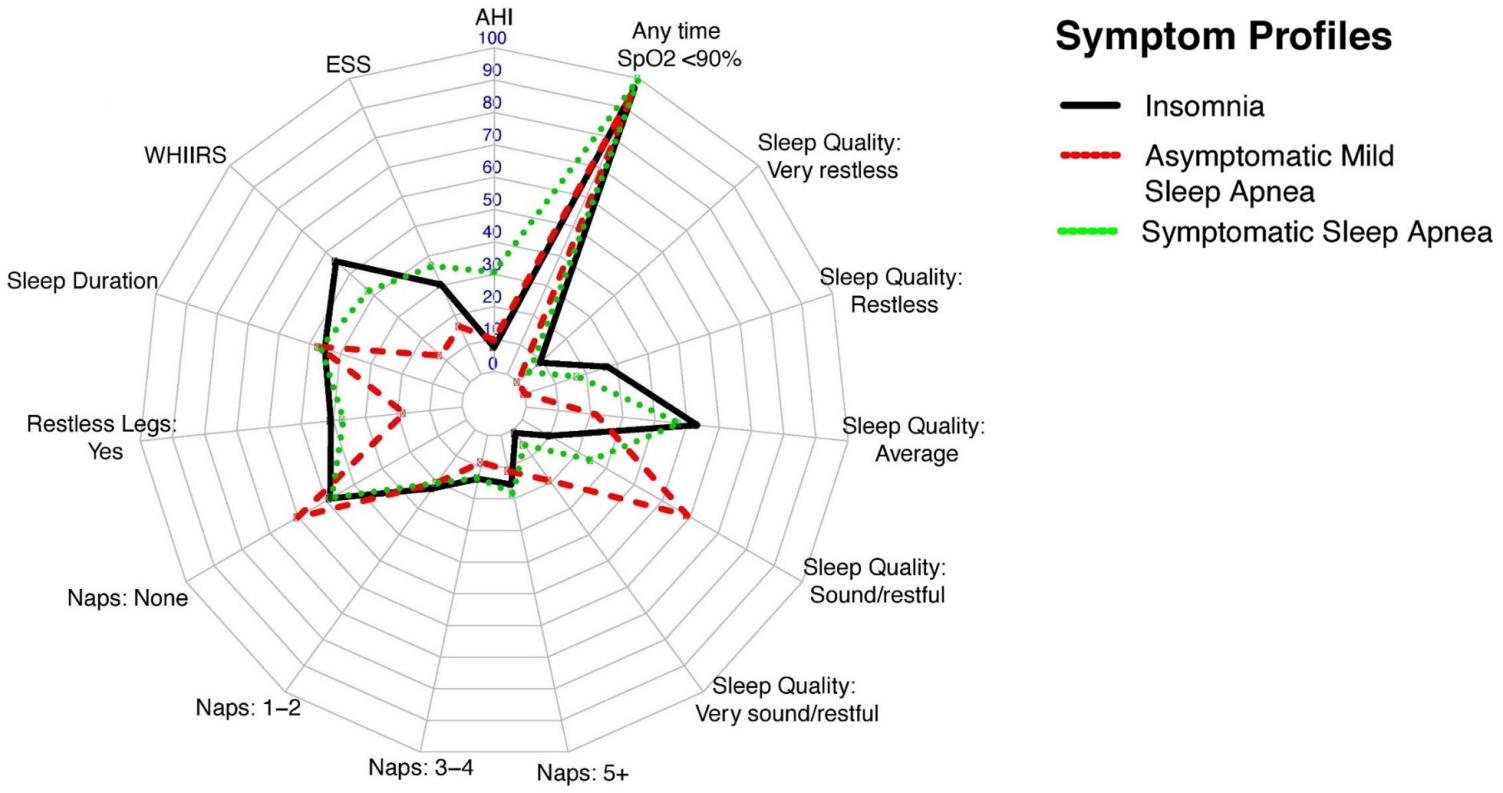
Symptom profile of primary latent class solution with HCHS/SOL individuals ages 45+ and AHI ≥5. All variables were rescaled to be on a 0–100 scale. Continuous indicators were rescaled using min-max normalization ([x-min(x)]/[max(x)-min(x)])*100. Categorical indicators are presented using the prevalence in % term.

We used seven sleep indicators, commonly assessed in clinical practice, and collected in HCHS/SOL (see above) to develop the latent classes. The LCA solutions were extracted sequentially (two to seven potential latent classes). In each model, the prevalence (for binary indicators) and the means and variances (for continuous measures) were unconstrained and assumed to vary across latent classes. The parameters in the LCAs were estimated using Full Information Maximum Likelihood to incorporate missingness and produce asymptotically unbiased estimates using all available data [43]. A Robust Maximum Likelihood Estimator was used to derive parameter estimates as well as inferential statistics (standard errors and chi-squared test statistics) that are robust to non-normality and non-independence of observations [44–46]. Participants were assigned to the class with the highest posterior probability following model adoption. The optimal adopted solution was determined by evaluating standard fit statistics (S1 and S2 Tables) for LCAs including Akaike Information Criterion (AIC), Bayesian Information Criterion (BIC), Vuong-Lo Mendel Rubin (LMR, VLMR), classification purity or entropy, class size, and clinical interpretability [25, 47, 48]. The estimated means (for the continuous sleep measures) and prevalence (for categorical indicators) for the derived phenotypes are presented in Table 1. To enable across group comparisons in estimated means and prevalence rates we also provide group specific comparisons in S3a and S3b Table. In line with previous work, we used data visualization to clarify classifications along the clinical indicators and facilitate the interpretation and efficiency in communicating information about each class composition (Fig 1). To generate the radar plot, all variables were rescaled to be on a 0–100 scale. Continuous indicators were rescaled using min-max normalization ([x-min(x)]/[max(x)-min(x)]) *100. Categorical indicators are presented using the prevalence in % terms.

**Table 1:** Symptom summary of HCHS/SOL individuals for three-group solution with survey design adjustment and subpopulation on Ages 45+ and AHI ≥5 (primary solution). Unweighted N = 3,545.

	Insomnia OSA	Asymptomatic Mild OSA	Symptomatic OSA	Total	*P* value
Unweighted N’s (Weighted %)	1596 (44.3%)	1275 (36.2%)	674 (19.5%)		
**AHI** *	10.5 (5.2)	13.8 (9.2)	43.5 (23.9)	18.1 (19.5)	*P*<0.001
**ESS** *	7.3 (6.2)	3.9 (3.6)	8.8 (7.4)	6.3 (6.1)	*P*<0.001
**WHIIRS** *	11.2 (5.5)	2.5 (2.2)	8.4 (6.0)	7.4 (6.6)	*P*<0.001
**Sleep Duration** *	7.7 (1.9)	7.9 (1.5)	7.9 (1.6)	7.8 (1.7)	*P* = 0.064
**Restless Legs** †					
No	59.2 (2.0)	82.1 (1.7)	63.2 (2.6)	68.3 (1.3)	*P*<0.001
Yes	40.8 (2.0)	17.9 (1.7)	36.8 (2.6)	31.7 (1.3)	
**Naps per week** †					
None	48.5 (2.1)	60.1 (2.2)	48.0 (2.7)	52.7 (1.4)	*P* = 0.001
1–2	22.3 (1.7)	20.1 (1.7)	20.3 (2.1)	21.1 (1.0)	
3–4	13.7 (1.6)	8.4 (0.9)	13.5 (2.2)	11.7 (0.9)	
5+	15.5 (1.8)	11.3 (1.5)	18.3 (1.9)	14.5 (1.0)	
**Sleep Quality** †					
Very sound/restful	1.1 (0.3)	19.4 (1.9)	5.7 (1.0)	8.7 (0.7)	*P*<0.001
Sound/restful	9.8 (1.1)	59.0 (2.3)	24.8 (2.2)	30.7 (1.3)	
Average	53.1 (2.1)	21.7 (1.8)	47.7 (2.7)	40.6 (1.3)	
Restless	26.9 (2.0)	0.0 (0.0)	17.2 (1.9)	15.2 (1.0)	
Very restless	9.0 (0.9)	0.0 (0.0)	4.6 (1.0)	4.8 (0.5)	
**Any time SpO2<90%** †					
No	3.3 (0.5)	4.0 (0.8)	0.1 (0.1)	2.9 (0.4)	*P*<0.001
Yes	96.7 (0.5)	96.0 (0.8)	99.9 (0.1)	97.1 (0.4)	

Notes:

* Means and Standard Deviations are presented;

^**†**^ % and Standard Errors (SEs) are presented

*P* value: Pearson’s chi square test for continuous variables; Regression based F test for categorical variables

**AHI**: Apnea-Hypopnea Index; **ESS**: Epworth Sleepiness Scale; **WHIIRS**: Women’s Health Initiative Insomnia Rating Scale; **SpO2**: Oxygen saturation

Second, we generated descriptive statistics to characterize the demographic and socioeconomic (Table 2) and cardiovascular (Table 3) profiles of the derived latent classes (OSA phenotypes). We used survey-adjusted chi-squared tests to examine overall differences in categorical variables, and survey-adjusted t-tests for continuous variables. As with above, to enable across group comparisons in estimated means and prevalence rates we also provide group specific comparisons in S4a, S4b, S5a and S5b Tables, respectively.

**Table 2:** Baseline sociodemographic and socioeconomic characteristics of HCHS/SOL individuals by primary solution of three derived sleep phenotypes.

	Insomnia OSA	Asymptomatic Mild OSA	Symptomatic OSA	Overall	*P* value	
Unweighted N’s (Weighted %)	1596 (44.3%)	1275 (36.2%)	674 (19.5%)			
**Age (years)** *	**58.0 (9.7)**	**58.4 (9.5)**	**58.0 (9.4)**	58.1 (9.6)	*P* = 0.623	**Legend**
**Sex** †						Lowest
Female	**56.3 (1.8)**	**39.0 (1.8)**	**36.6 (2.3)**	46.2 (1.1)	*P*<0.001	
Male	**43.7 (1.8)**	**61.0 (1.8)**	**63.4 (2.3)**	53.8 (1.1)		Highest
**Race/Ethnicity** †						
Central American	**4.9 (0.6)**	**7.0 (0.8)**	**5.1 (0.8)**	5.7 (0.5)	*P*<0.001	
Cuban	**25.5 (2.4)**	**30.6 (3.1)**	**32.8 (2.8)**	28.8 (2.2)		
Dominican	**9.8 (1.3)**	**6.4 (0.9)**	**8.9 (1.6)**	8.4 (0.8)		
Mexican	**28.8 (1.9)**	**36.5 (2.7)**	**28.5 (2.7)**	31.6 (1.9)		
Puerto Rican	**23.5 (2.2)**	**10.6 (1.1)**	**18.7 (2.1)**	17.9 (1.3)		
South American	**5.1 (0.6)**	**5.6 (0.8)**	**4.2 (0.8)**	5.1 (0.5)		
Other	**2.3 (0.5)**	**3.3 (0.7)**	**1.6 (0.6)**	2.5 (0.3)		
**Education** †						
<12 years	**44.5 (2.0)**	**39.0 (2.2)**	**39.9 (2.6)**	41.6 (1.4)	*P* = 0.259	
12 years	**18.5 (1.3)**	**21.2 (1.6)**	**21.5 (2.1)**	20.1 (1.0)		
>12 years	**37.0 (2.0)**	**39.8 (2.1)**	**38.6 (2.5)**	38.3 (1.3)		
**Income** †						
<$30,000	**68.3 (1.9)**	**61.9 (2.3)**	**63.4 (2.7)**	65.0 (1.5)	*P* = 0.055	
> = $30,000	**24.6 (1.7)**	**31.6 (2.2)**	**30.9 (2.6)**	28.4 (1.4)		
Not reported	**7.1 (1.0)**	**6.5 (1.1)**	**5.7 (1.4)**	6.6 (0.7)		
**Occupation Longest** †						
Non-skilled worker	**25.0 (1.8)**	**24.5 (2.0)**	**22.1 (1.9)**	24.3 (1.2)	*P* = 0.202	
Service worker	**13.3 (1.2)**	**16.0 (1.7)**	**13.2 (1.8)**	14.2 (0.9)		
Skilled worker	**23.5 (2.0)**	**19.1 (1.6)**	**25.8 (2.5)**	22.3 (1.2)		
Professional/technical/other office worker	**15.7 (1.3)**	**19.4 (1.8)**	**15.7 (2.0)**	17.0 (1.1)		
Other	**22.5 (1.9)**	**21.0 (1.7)**	**23.2 (2.2)**	22.1 (1.1)		
**Occupation current + Employment status** †						
Non-skilled worker	**9.5 (1.0)**	**12.9 (1.3)**	**8.0 (1.4)**	10.4 (0.7)	*P* = 0.002	
Service worker	**7.9 (1.0)**	**8.8 (1.0)**	**11.6 (1.6)**	8.9 (0.7)		
Skilled worker	**7.8 (0.8)**	**10.1 (1.2)**	**10.8 (2.0)**	9.3 (0.7)		
Professional/technical/other office worker	**4.4 (0.7)**	**7.6 (1.1)**	**3.8 (0.8)**	5.4 (0.5)		
Other	**7.8 (1.0)**	**6.3 (0.8)**	**8.4 (1.5)**	7.4 (0.6)		
Retired & Not employed	**24.1 (2.0)**	**24.3 (2.1)**	**24.9 (2.4)**	24.4 (1.3)		
Not retired & not employed	**38.6 (2.0)**	**29.9 (1.9)**	**32.4 (2.4)**	34.2 (1.3)		
**SASH Language subscale** *	**1.8 (1.2)**	**1.6 (1.0)**	**1.8 (1.2)**	1.8 (1.2)	*P*<0.001	
**SASH Social subscale** *	**2.2 (0.8)**	**2.1 (0.7)**	**2.2 (0.7)**	2.2 (0.7)	*P* = 0.015	

Notes:

Unweighted N = 3,545.

* Means and Standard Deviations are presented;

^**†**^ % and Standard Errors (SEs) are presented

*P* value: Pearson’s chi square test for continuous variables; Regression based F test for categorical variables

**HS**: High School; **SASH**: Short Acculturation Scale for Hispanics

The legend indicates which sleep phenotype groups have the highest and lowest values for each variable.

**Table 3:** Baseline cardiovascular characteristics of HCHS/SOL individuals by primary solution derived sleep phenotype.

	Insomnia OSA	Asymptomatic Mild OSA	Symptomatic OSA	Total	*P* value	
Unweighted N’s (Weighted %)	1596 (44.3%)	1275 (36.2%)	674 (19.5%)			
**HDL Cholesterol (mg/dL)⇞**	**49.1 (14.7)**	**47.7 (15.9)**	**44.0 (12.4)**	47.6 (14.9)	*P*<0.001	**Legend**
**Total Cholesterol (mg/dL)⇞**	**206.5 (53.1)**	**209.0 (50.0)**	**205.8 (55.3)**	207.3 (52.5)	*P* = 0.489	Lowest
**Triglycerides (mg/dL)⇞**	**149.8 (104.6)**	**158.8 (130.0)**	**175.3 (157.3)**	158.1 (126.5)	*P* = 0.001	Medium
**BMI (kg/m2)⇞**	**31.4 (6.7)**	**30.6 (5.9)**	**33.7 (7.0)**	31.6 (6.6)	*P*<0.001	Highest
**Cigarette Usage** †						
Never	**54.1 (2.0)**	**55.4 (1.9)**	**44.9 (2.6)**	52.8 (1.3)	*P* = 0.032	
Former	**28.5 (1.6)**	**28.7 (1.7)**	**33.9 (2.6)**	29.6 (1.1)		
Current	**17.4 (1.6)**	**15.9 (1.4)**	**21.1 (2.4)**	17.6 (1.0)		
**Alcohol Usage** †						
Doesn’t drink alcohol	**55.4 (1.8)**	**53.6 (2.0)**	**57.5 (2.4)**	55.2 (1.2)	*P* = 0.459	
Drinks alcohol	**44.6 (1.8)**	**46.4 (2.0)**	**42.5 (2.4)**	44.8 (1.2)		
**CVD**						
No CVD	**49.2 (2.0)**	**63.2 (1.9)**	**47.4 (2.7)**	54.0 (1.3)	*P*<0.001	
CVD	**50.8 (2.0)**	**36.8 (1.9)**	**52.6 (2.7)**	46.0 (1.3)		
**Heart Failure**						
No heart failure	**96.4 (0.7)**	**97.5 (0.8)**	**95.9 (1.0)**	96.7 (0.4)	*P* = 0.410	
Heart failure	**3.6 (0.7)**	**2.5 (0.8)**	**4.1 (1.0)**	3.3 (0.4)		
**Stroke/TIA** †						
No Prevalent Stroke/TIA	**94.1 (0.8)**	**94.6 (1.1)**	**95.8 (0.9)**	94.6 (0.5)	*P* = 0.541	
Prevalent Stroke/TIA	**5.9 (0.8)**	**5.4 (1.1)**	**4.2 (0.9)**	5.4 (0.5)		
**Hypertension** †						
Not hypertensive	**43.7 (2.0)**	**43.7 (2.1)**	**31.8 (2.4)**	41.4 (1.2)	*P* = 0.001	
Hypertensive	**56.3 (2.0)**	**56.3 (2.1)**	**68.2 (2.4)**	58.6 (1.2)		
**Diabetes** †						
Non-diabetic	**66.1 (1.9)**	**66.6 (2.0)**	**57.3 (2.7)**	64.6 (1.3)	*P* = 0.012	
Diabetic	**33.9 (1.9)**	**33.4 (2.0)**	**42.7 (2.7)**	35.4 (1.3)		
**FRS Score**	**0.14 (0.13)**	**0.15 (0.13)**	**0.20 (0.15)**	0.11 (0.12)	*P*<0.001	
**FRS Score (3 categories)**						
<0.1	**37.2 (2.0)**	**27.4 (1.7)**	**21.0 (1.9)**	30.5 (1.2)	*P*<0.001	
0.1-<0.2	**32.3 (2.0)**	**35.5 (2.0)**	**34.3 (2.6)**	33.9 (1.1)		
> = 0.2	**30.4 (1.9)**	**37.1 (2.2)**	**44.7 (2.8)**	35.6 (1.3)		

Notes:

Unweighted N = 3,545.

* Means and Standard Deviations are presented;

^**†**^ % and Standard Errors (SEs) are presented

*P* value: Pearson’s chi square test for continuous variables; Regression based F test for categorical variables

**HDL**: High-density lipoproteins; **BMI**: Body Mass Index: Transient Ischemic Attack

The legend indicates which sleep phenotype groups have the highest and lowest values for each variable.

**Fig 2:**
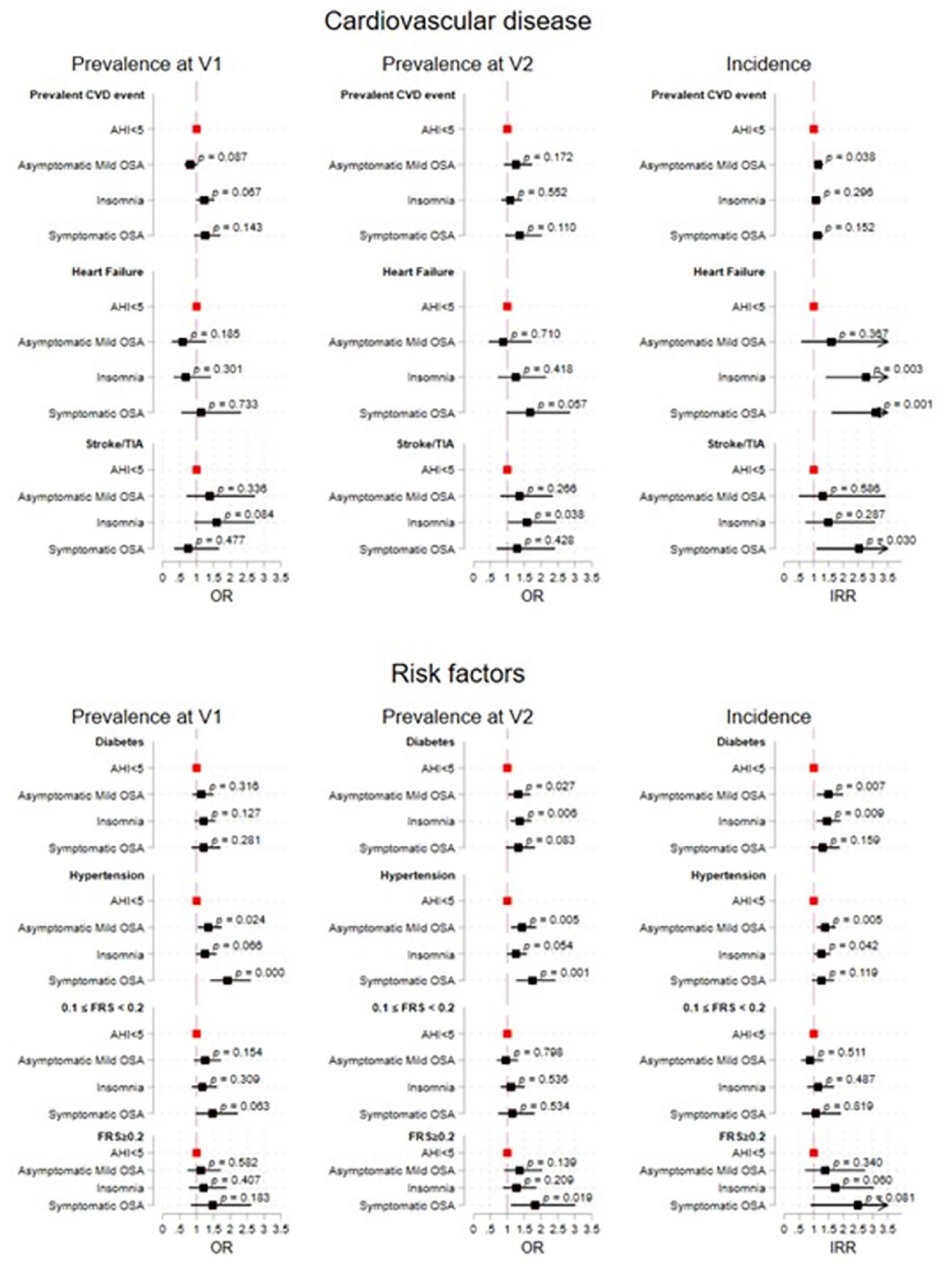
The estimated parameters and inferential statistics for the associations of OSA phenotypes with the cardiovascular disease and risk outcomes. AHI<5 is the reference group. The prevalence at V1 models are adjusted for BMI, cigarette usage, alcohol usage, HDL cholesterol, total, cholesterol, and triglycerides all at V1. The prevalence at V2 models are adjusted for BMI, cigarette usage, alcohol usage, HDL cholesterol, total, cholesterol, and triglycerides all at V2. The incidence models are adjusted for BMI, cigarette usage, alcohol usage, HDL cholesterol, total, cholesterol, and triglycerides all at V1.

Third, we fit survey logistic regression models to examine the associations between OSA phenotypes (as an exposure) and prevalence of cardiovascular risk factors and disease at Visit 1 and Visit 2. We then used survey Poisson regression to examine associations between OSA phenotypes and incidence of cardiovascular risk factors and disease between Visit 1 and Visit 2. Incidence analyses focused on the subpopulation of individuals without the reported cardiovascular risk/event at baseline. For all logistic and Poisson analysis, we set the low-risk AHI <5 group as the reference category. Descriptive statistics comparing the sociodemographic, socioeconomic, and cardiovascular characteristics of the OSA phenotypes to individuals who had an AHI<5 are presented in S6 Table. For both the survey logistic and Poisson regression, we fit three models: (1) crude (without covariates adjustments), (2) age, sex, and Latino background, and (3) age, sex, and Latino background, BMI, cigarette usage, alcohol usage, HDL cholesterol, total cholesterol, and triglycerides. The estimated parameters and inferential statistics for the associations of OSA phenotypes with the cardiovascular outcomes are found in S7 Table and visualized in Fig 2.

### Sensitivity analyses

We conducted two sets of sensitivity analyses (S1, and S2). First, the LCA models were re-estimated including adjustments for income, language acculturation, social acculturation, sex, and age to assess whether and to what extent these indicators bias the derived classifications. Then, we re-estimated the LCA models in the overall subpopulation of participants ages 45-years and older including individuals with AHI<5. The fit statistics for the sensitivity LCA models are presented in S8/S8a Table. The characterization of the adopted class solutions through means (for the continuous sleep measures) and prevalence estimates (for categorical indicators) for the derived phenotypes are presented in S9 and S10 Tables and visualized in S2 Fig.

All LCAs were performed using MPLUS Version 8.3. Descriptive analyses and survey adjusted tests for group differences were done in Stata V.16. Visualizations of class solutions were performed using R.3.6.3 using the *ggplot* package. Regression analyses were performed using Stata 16. All models and tests accounted for the complex survey design of the HCHS/SOL to allow appropriate generalization to the target population of interest.

## Results

### Characteristics of the target population

The average age of the target subpopulation (i.e., AHI>5) was 58.1±9.6 years (Mean ± Standard Deviations; SD), 46.2% were females, 41.6% had less than 12-years of education, about two-thirds reported a household income less than $30,000 a year, and three in five reported having a non-skilled/service/or skilled work their longest occupation.

### Latent class solution

Based on assessment of fit statistics (AIC, BIC, entropy, VLMR, and LMR tests) the three-class solution provided best fit to the data and the most meaningful clinical interpretation. The fit statistics are summarized in S1 Table. The three OSA phenotypes were labeled as (1) ***insomnia OSA*** (44.3%), (2) ***asymptomatic mild OSA*** (36.2%), and (3) ***symptomatic OSA*** (19.5%). The prevalence and symptoms characteristics of the sleep measures are listed in Table 1 and are visualized in Fig 1.

The ***insomnia OSA*** group had an average AHI of 10.5 events/hours (Standard error; SE = 5.2); the mean ESS, WHIIRS, and sleep duration were 7.3 (SE = 6.2), 11.2 (SE = 5.5), and 7.7 hours (SE = 1.9), respectively (Table 1). Every 2 in 5 individuals of the target population (40.8%) reported restless legs symptoms, 15.5% reported 5+ naps per week (near daily) naps of more than 5 minutes, and 35.9% had low quality sleep (restless or very restless) over the past 4-weeks, and only 3.3% had no time where SpO2 < 90%. The ***asymptomatic mild OSA*** group had an average AHI of 13.8 (SE = 9.2). Mean ESS and WHIIRS were low; 3.9 (SE = 3.7), and 2.5 (SE = 2.2), respectively. Less than a fifth (17.9%) reported restless legs symptoms, 11.3% reported near daily naps of more than 5 minutes, none reported low quality sleep (restless or very restless) over the past 4-weeks, and 4.0% had no time where SpO2 < 90%. The ***symptomatic OSA*** group had an average AHI of 43.5 (SE = 24.1). Mean ESS and WHIIRS were 8.8 (SE = 7.4), and 8.4 (SE = 6.0), respectively. Slightly more than a third (36.8%) reported restless legs symptoms, 18.3% reported near daily naps, slightly more than 21.8% had low quality sleep (restless or very restless) over the past 4-weeks, and nearly all had time where SpO2 < 90%.

### Demographic characteristics of OSA phenotypes (Table 2)

Males were more likely to be classified in the ***asymptomatic mild OSA*** (61.0%) and ***symptomatic OSA*** (63.4%) groups whereas the ***insomnia OSA*** group had a much higher female composition (56.3%). The ***insomnia OSA*** group was the most likely to have less than 12-years of education (44.5%) and most likely to report a yearly household income <$30,000 (68.3%). Additionally, 38.6% in the ***insomnia OSA*** group reported being currently unemployed. We found no substantive or statistical differences in the age and acculturation profiles of the latent OSA phenotypes.

### Cardiovascular risk factors and vascular disease profile (Table 3)

The ***insomnia OSA*** group had the highest average HDL cholesterol and were most likely to have prevalent stroke/TIA out of the three groups. The individuals in this group also had the second highest average total cholesterol and BMI. The ***asymptomatic mild OSA*** group had the highest total cholesterol and were most likely to report alcohol consumption. However, they were the less likely to report cardiovascular diseases including hypertension, and diabetes. The ***symptomatic OSA*** group had the worst cardiovascular health profile relative to the insomnia and asymptomatic mild OSA groups. Individuals in this group had the highest average triglycerides and BMI. They were also most likely to be former or current smokers and have prevalent CVD events, heart failure, hypertension, and diabetes.

### Prevalent cardiovascular outcomes (S7 Table, Fig 2)

Compared to participants without OSA, the ***asymptomatic mild OSA*** group had higher odds ratios for hypertension prevalence at Visit 1 (OR = 1.35 [1.04;1.74], p<0.05) and Visit 2 (1.44 [1.12;1.85], p<0.01) and for diabetes prevalence at Visit 2 (OR = 1.32 [1.03;1.68], p<0.05). We also found higher odds ratios in the ***insomnia OSA*** group for prevalent diabetes (OR = 1.37 [1.10;1.72], p<0.01) and stroke (OR = 1.58 [1.02;2.45], p<0.05) at Visit 2 when compared to participants without OSA. Lastly, the ***symptomatic OSA*** group had higher odds for prevalent hypertension at Visit 1 (OR = 1.92 [1.42;2.60], p<0.001) and Visit 2 (1.75 [1.27;2.43], p<0.001), and for prevalent diabetes (OR = 1.39 [1.02;1.90], p<0.05), and stroke at Visit 2 (OR = 1.32 [0.96;1.82], p = 0.083; marginal significance) relative to individuals without OSA.

### Incident cardiovascular outcomes (S7 Table, Fig 2)

***Asymptomatic mild OSA*** individuals had higher incident risk ratios (IRR) for CVD events (IRR = 1.15[1.01;1.31], p<0.05), hypertension (IRR = 1.39 [1.11;1.74], p<0.01) and diabetes (IRR = 1.49 [1.11;1.99], p<0.01) relative to those without OSA. Additionally, individuals satisfying criteria for the ***insomnia OSA*** phenotype had elevated incidence of heart failure (IRR = 2.76 [1.41;5.39], p<0.01), diabetes (IRR = 1.44 [1.10;1.91], p<0.01) and hypertension (IRR = 1.25 [1.01;1.56], p<0.05) relative to those without OSA. Lastly, the ***symptomatic OSA*** group had higher incidence of heart failure (IRR = 3.11 [1.60;6.03], p<0.001) and stroke (IRR = 2.53 [1.10;5.83], p<0.05) compared to individuals without OSA.

### Sensitivity results

The first sensitivity analysis included re-estimating the LCA adjusting for income, language acculturation, social acculturation, sex, and age to verify stability and robustness of the three identified groups in this sample. There were no marked differences that altered the identified phenotype cluster definitions or distributions of sociodemographic and cardiovascular risk factors and disease across groups. We ran an additional sensitivity focusing on individuals ages 45-years and older independent of AHI index score. Under this scenario we derived four phenotypes including (1) an ***insomnia*** group (25.9%), (2) an ***asymptomatic with mild OSA*** group (25.3%), (3) ***symptomatic OSA*** group (19.1%), and (4) an ***asymptomatic*** group (29.8%). As expected, due to stratification on mild AHI criteria in the primary solution, the derived phenotypes under the sensitivity scenario validates the three derived phenotypes reported in the primary analyses.

## Discussion

Our study is the first to identify clusters of OSA phenotypes in community dwelling, middle aged and older, diverse Hispanic/Latinos in the US. Our results offer insight on the distribution of OSA symptoms, clinical severity, and associated sleep symptoms (e.g. insomnia) using a novel methodological application and a data-driven approach. Two primary findings emerge. First, we report three clinically meaningful OSA phenotypes: (1) an insomnia OSA group, (2) an asymptomatic mild OSA group, (3) and a symptomatic OSA group. Second, distinct sociodemographic and cardiovascular measures characterized phenotypes of particular significance to Hispanic/Latino populations. In line with previous work, our results point to a heterogeneous presentation that can capture the differing experiences of individuals with OSA. Better identification of these phenotypes creates opportunities to develop personalized approaches for investigating and treating OSA. Of interest, the symptomatic OSA group had increased prevalence and incident stroke. While OSA has been shown to be a risk factor for stroke and CVD, treatment of OSA has not consistently reduced vascular risk in clinical trials; results were partly explained by suboptimal adherence to positive airway pressure therapy [49]. However, our findings suggest that identifying at risk individuals using OSA phenotypes, could lead to personalized care and targeted clinical trials for risk reduction of stroke [50, 51]. Sleep metrics, such as sleep duration, insomnia symptoms and restless legs have been associated with stroke and CVD risk [52–54]. However, most studies do not account for other sleep confounders or the co-occurrence of sleep disorders. Our analysis accounts for multiple sleep metrics, providing a novel and robust approach to examine at risk-individuals for sleep related adverse health outcomes.

In our sample, the asymptomatic mild OSA group also had increased incident CVD. In addition, the insomnia-OSA group and the symptomatic OSA-group had strong associations with heart failure. In line with our findings, an analysis of the Sleep Heart Health Study conducted LCA with 14 sleep symptoms, plus the ESS. This population-based study described four OSA phenotypes: 1) Disturbed sleep, 2) Minimally Symptomatic, 3) Excessively Sleepy, and 4) Moderately Sleep [24]. This study observed that individuals in the excessively sleepy (average ESS was 13.7) OSA group were most likely to be males with a higher BMI and increased cardiovascular risk factors (e.g. diabetes), when compared to individuals in the minimally or asymptomatic group [13]. In addition, the sleepy group, had strong associations with prevalent and incident CVD and heart failure [24]. The OSA phenotypes derived in our study had relatively normal daytime sleepiness <8.8 and were primarily characterized by the AHI, insomnia, and OSA symptoms. These findings underscore the potential for data-driven techniques to identify more refined targets for interventions and development of precision medicine and treatment for shared morbidity clusters.

In our study, a large proportion of the OSA patients that clustered in the insomnia group endorsed restless legs symptoms (RLS) and low sleep quality. Importantly, there is a paucity of data on restless legs symptoms and its consequences in Hispanic/Latino populations. Of interest, the prospective multi-center Determining Risk of Vascular Events by Apnea monitoring (DREAM) study [23], described seven different clusters using polysomnography. The cluster of OSA with periodic limb movements in sleep, which are seen in up to 80% of RLS patients, had a hazard ratio of 2.4 [95%CI = 1.6–3.5] for cardiovascular events and mortality. OSA categories based on the clinical AHI cutoffs did not predict vascular outcomes [23]. In a different study, RLS symptoms (without OSA) was associated with increased CVD related mortality compared to the reference group [52].

We also observed that individuals in the symptomatic OSA-group, as well as those in the asymptomatic OSA group, had increased prevalent hypertension and diabetes mellitus at HCHS/SOL baseline and Visit 2. Like our findings, an analysis of 6,965 hypertension free HCHS/SOL participants at visit 1, insomnia (with a normal AHI) and OSA (AHI ≥ 5) predicted incident hypertension after a median follow-up of 6.1 [SD: 0.8] years [55]. We extended these findings by evaluating associations between the OSA phenotypes with prevalence of cardiovascular risk factors and disease at both Visits one and two, as well as incidence of cardiovascular risk factors and disease. Most observational studies observe worse outcomes in participants with daytime sleepiness associated with OSA. Asymptomatic individuals are less likely to be referred for evaluation and treatment of a sleep disorder, which could expose the detrimental effects of OSA and its associated CVD comorbidities for prolonged periods [50, 51, 56]. Traditional CPAP treatment has been found to not be associated with a lower rate of major adverse cardiovascular and cerebrovascular events [57]. However, there are some surgical interventions that are available, such as barbed reposition pharyngoplasty and expansion sphincter pharyngoplasty to treat OSA [58, 59].

Consistent with previous publications, our results showed a heterogeneity of OSA phenotypes beyond the standard AHI criteria [19–23, 25, 38, 48]. For example, the Icelandic Sleep Apnea Cohort (*n* = 822) of patients with moderate to severe sleep apnea (AHI ≥ 15) described three different phenotypic clusters: insomnia, minimal symptoms and excessive daytime sleepiness [21]. The Sleep Apnea Global Interdisciplinary Consortium (SAGIC) study confirmed and extended these results by also including patients from an international sample [25]. Interestingly, SAGIC showed that females and minorities had more “disturbed sleep” than other cluster types, in addition to decreased compliance to positive airway pressure therapy [13, 24].

We also observed marked sex differences in the distribution of our clusters [60]. Individuals with the Insomnia OSA phenotype were more likely to be females and reported the lowest levels of education and household income, and non-skilled worker as their longest occupation and/or currently unemployed compared to the other groups. Interestingly, we found no differences in social and language acculturation across the three phenotypes. Future work should also use multidomain classifications with a richer set of combined sleep symptoms, health endophenotypes, and treatment modalities to validate the stability of these clusters and underscore the potential effects of treatment on risk reduction.

### Strengths and limitations

Our study is the first to identify OSA phenotypes in diverse Hispanic/Latinos using a large and representative sample of community dwelling middle-aged and older adults with systematic assessments of their social, behavioral, health, and sleep characteristics. Existing phenotypic clustering studies are predominantly based on middle-aged and older Whites and are largely composed of individuals presenting to sleep clinics or with moderate and high levels of OSA severity. This group did not reflect the heterogeneity of OSA and its symptomatic presentation, particularly given known context [61] and other determinants for these symptoms [62]. We use extensive modeling to validate the stability of classifications in a higher risk group including individuals with mild/moderate and severe OSA. The additional models also confirmed our classification after adjustments for important covariates and provided evidence of robustness to these potential confounds. OSA is a risk factor for stroke and cardiovascular outcomes that are disparately prevalent among Hispanics/Latinos [38]. Future longitudinal work may explore distal cognitive outcomes and mortality as well with our OSA phenotypes.

Our results should also be interpreted in the context of several limitations. First, the home sleep apnea test does not include measures of electroencephalography (EEG) or leg movements, preventing evaluation of arousal, sleep architecture, and periodic limb movements. We also selected a brief subset of measures that are both relatively easy to obtain during clinical encounters and clinically meaningful and help maintain concordance with available literature on sleep risks for critical prospective outcomes (particularly cardiovascular disease and cognition). The potential causal pathways underlying these disorders cannot be assessed with our study design. Second, there could be reporting bias particularly for self-reported sleep data. The large sample size and standardized assessments adds confidence, in that any biases are likely unsystematic. Confounding through unmeasured factors is still possible in any observational study such as this one. Factors such as low health literacy, low medication usage, and low awareness of comorbid conditions might have had an impact on our results as unmeasured confounders. Third, HCHS/SOL was designed to address gaps in scientific knowledge on U.S. Hispanic/Latino health. The derived sleep phenotypes in this study should be validated using other cohorts, including other race/ethnic groups, given differing risk factors for OSA and CVD across subpoulations [63]. Fourth, there is potential classification error when using LCA models, given that individuals are assigned to their highest probability class which can lead to uncertainty in classification. We conducted several sensitivity analyses to ensure that classifications resulting from our models were stable. However, future work is needed to validate the stability of the identified classes and their characteristics. Finally, estimates of the prevalence and incidence rates of cardiovascular outcomes may have large variances due to the sample size given our desired subpopulation. HCHS/SOL is in the process of collecting a third wave of data on cohort participants, including cardiovascular data. Future work should replicate our results using these data as the prevalence and incidence of cardiovascular disease are expected to increase due to the natural aging of the cohort.

## Conclusion

We identified three groups using LCA techniques in a community-based sample of diverse U.S. Hispanic/Latino adults: 1) Insomnia OSA, 2) Asymptomatic mild OSA, and 3) Symptomatic OSA. The three-identified groups each had different cardiovascular and sociodemographic characteristics. The three-identified groups also had varying risk of prevalent and incident cardiovascular risk factors and disease. Our results point to future opportunities for assessing multidimensional risks faced by Hispanics/Latinos, specifically at the intersection of sleep and important outcomes such as neurocognitive decline. Furthermore, we suggest that our approach be applied to other ethnic/racial groups for tailoring sleep phenotyping [30]. Better characterization of OSA phenotypes for Hispanics/Latinos can help in developing new research approaches and targeted interventions studies to ameliorate health disparities.

## Supporting information

S1 Fig Schema for analytical sample. (TIF)

S2 Fig Symptom profile of latent class solution with HCHS/SOL individuals ages 45+. (TIF)

S1 Table Latent Class Analysis model fit statistics of sleep phenotypes. Models are adjusted for survey design and subpopulated on HCHS/SOL individuals ages 45+ and with AHI ≥5. Unweighted N = 3,545.
(DOCX)

S2 Table Latent Class Analysis model fit statistics. Models are not adjusted for survey design and subpopulated on HCHS/SOL individuals ages 45+ and with AHI ≥5. N = 3,545.
(DOCX)

S3 Table a. Symptom comparisons across sleep phenotypes relative to Asymptomatic with Mild OSA group for the primary solution. b. Symptom means and proportion contrasts across sleep phenotypes for the primary solution.
(DOCX)

S4 Table a. Socioeconomic and sociodemographic characteristics comparisons across sleep phenotypes relative to Asymptomatic with Mild OSA group for the primary solution. b. Socioeconomic and sociodemographic means and proportion contrasts across sleep phenotypes for the primary solution.
(DOCX)

S5 Table a. Cardiovascular characteristics comparisons across sleep phenotypes relative to Asymptomatic with Mild OSA group for the primary solution. b. Cardiovascular means and proportion contrasts across sleep phenotypes for the primary solution.
(DOCX)

S6 Table Sociodemographic, socioeconomic, and cardiovascular characteristics of HCHS/SOL individuals for three-group solution and AHI<5 individuals with survey design adjustment and subpopulation on Ages 45+. Unweighted N = 5,588.
(DOCX)

S7 Table Associations between OSA phenotypes and incidence of cardiovascular risks. (DOCX)

S8 Table Latent class analysis model fit statistics. Models are adjusted for survey design and subpopulated on HCHS/SOL individuals ages 45+. a. Latent class analysis model fit statistics. Models are not adjusted for survey design and subpopulated on HCHS/SOL individuals ages 45+. Unweighted N = 9,617.
(DOCX)

S9 Table Symptom summary of HCHS/SOL individuals for four-group solution with survey design adjustment and subpopulation on ages 45+ (supplementary solution). Unweighted N = 9,617.
(DOCX)

S10 Table Baseline sociodemographic, socioeconomics, and cardiovascular characteristics of HCHS/SOL individuals by supplementary solution derived sleep phenotypes. (DOCX)
