# Supplementary figures and images for: Cardiovascular correlates of sleep apnea phenotypes: Results from the Hispanic Community Health Study/Study of Latinos (HCHS/SOL)
Source: PLoS One. 2022 Apr 4;17(4):e0265151. doi: 10.1371/journal.pone.0265151 (PMC8979447; doi:10.1371/journal.pone.0265151)

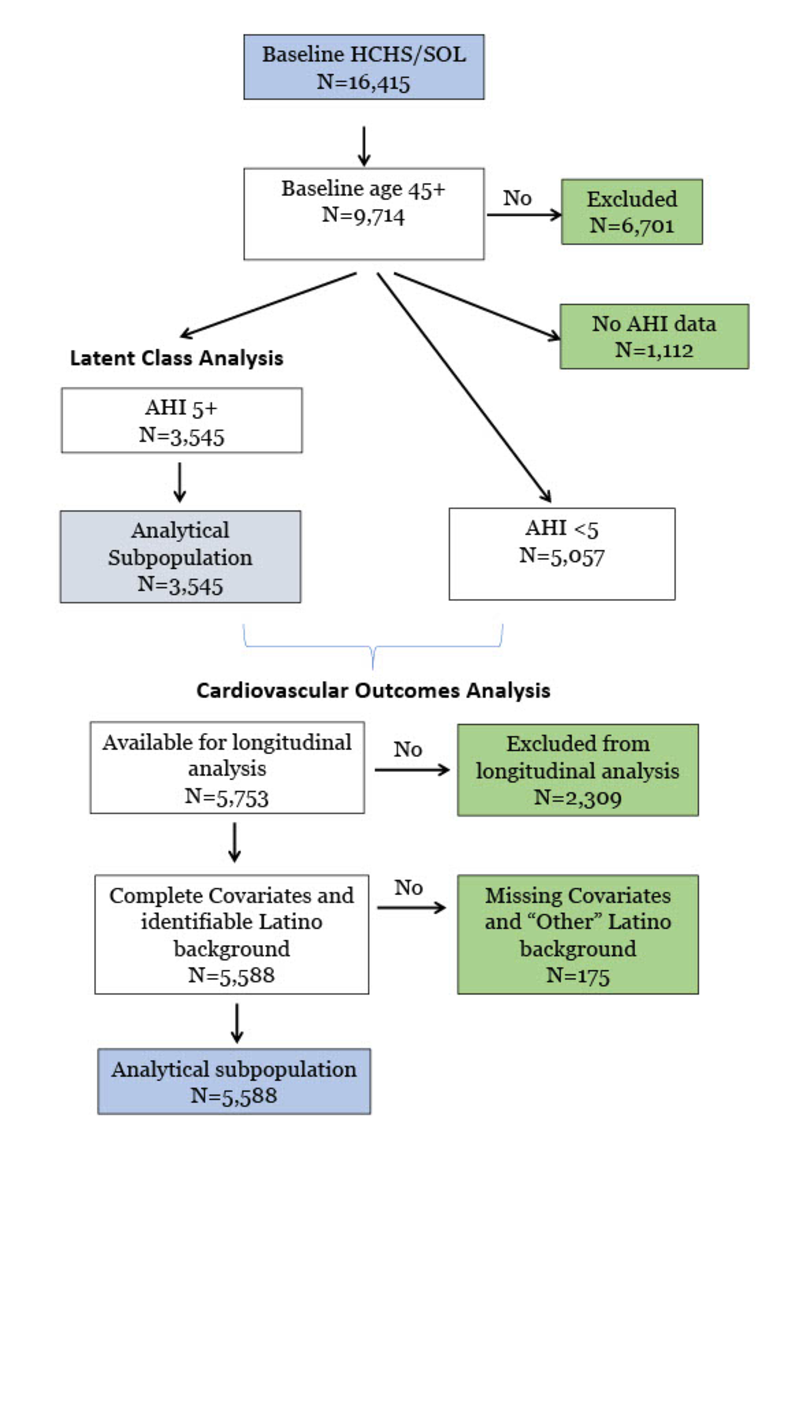

Supplement: S1 Fig — (TIF) [file pone.0265151.s001.tif]

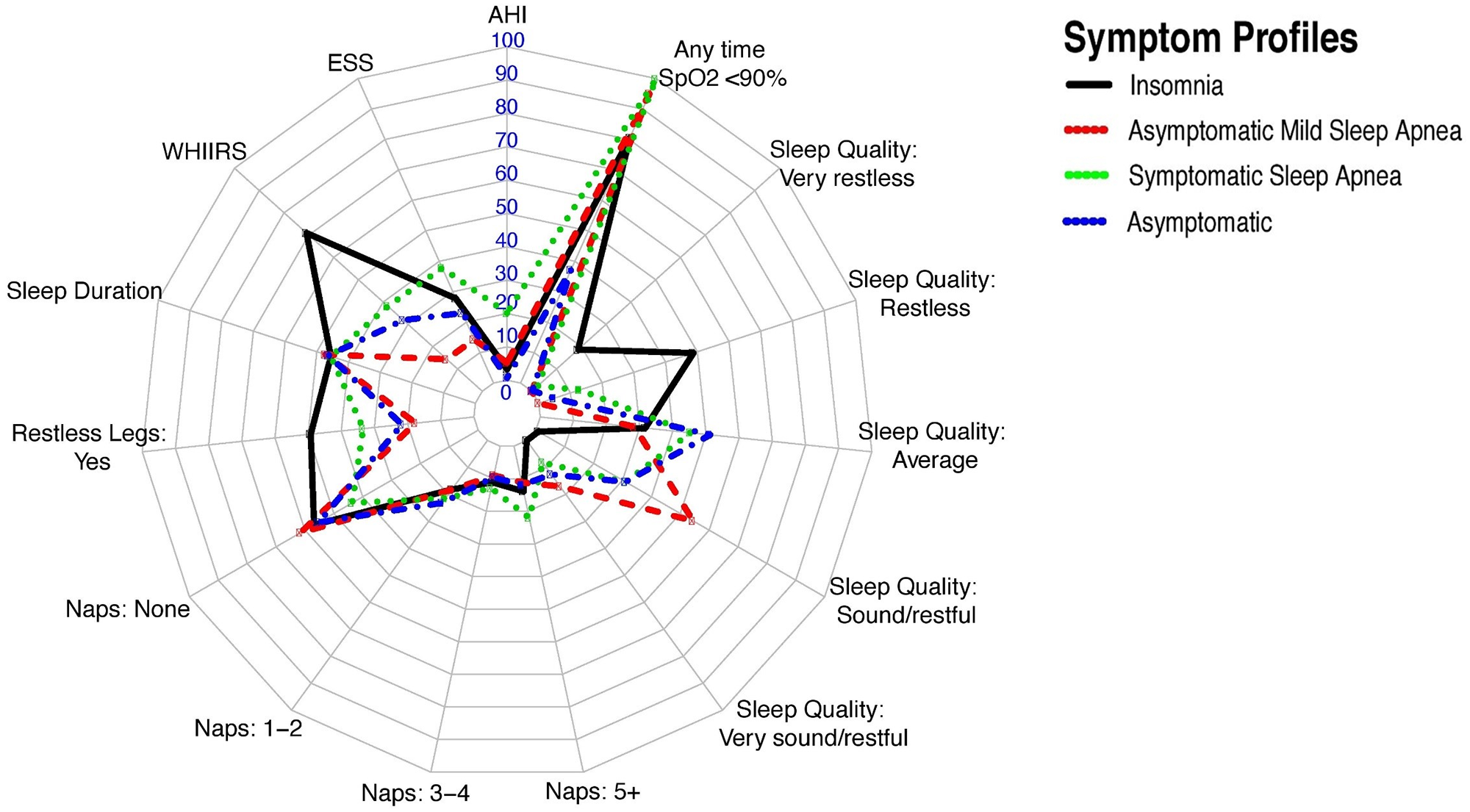

Supplement: S2 Fig — (TIF) [file pone.0265151.s002.tif]
