# Supplementary material for: Cardiovascular correlates of sleep apnea phenotypes: Results from the Hispanic Community Health Study/Study of Latinos (HCHS/SOL)
Source: PLoS One. 2022 Apr 4;17(4):e0265151. doi: 10.1371/journal.pone.0265151 (PMC8979447; doi:10.1371/journal.pone.0265151)
Supplement: S1 Table — Models are adjusted for survey design and subpopulated on HCHS/SOL individuals ages 45+ and with AHI ≥5. Unweighted N = 3,545. (DOCX) [file pone.0265151.s003.docx]

**S1 Table.** **Latent Class Analysis model fit statistics of sleep phenotypes. Models are adjusted for survey design and subpopulated on HCHS/SOL individuals ages 45+ and with AHI ≥5. Unweighted N=3,545.**

| **Solution** | **LL** | **Scaling Correction Factor** | **Free Parameters** | **AIC** | **BIC** | **SSABIC** | **Entropy** | **VLMR *P* value** | **LMR *P* value** | **VLMR *P* value*** | **LMR *P* value*** | **AICc** |
| --- | --- | --- | --- | --- | --- | --- | --- | --- | --- | --- | --- | --- |
| C2 | -52174.075 | 2.9895 | 35 | 104418.149 | 104634.214 | 104523.002 | 0.723 | 0.0118 | 0.0121 | <0.0000 | <0.0000 | 104418.8672 |
| C3 | -51045.007 | 2.7126 | 53 | 102196.014 | 102523.198 | 102354.791 | 0.742 | 0.0355 | 0.0366 | 0.0720 | 0.0729 | 102197.6536 |
| C4 | -50513.452 | 2.9321 | 71 | 101168.903 | 101607.207 | 101381.605 | 0.770 | 0.3257 | 0.3282 | 0.0636 | 0.0645 | 101171.8469 |
| C5 | -50160.836 | 2.7438 | 89 | 100499.672 | 101049.095 | 100766.298 | 0.748 | 0.2576 | 0.2593 | 0.0019 | 0.0020 | 100504.3088 |
| C6 | -49910.458 | 2.5272 | 107 | 100034.915 | 100695.458 | 100355.466 | 0.750 | 0.3817 | 0.3831 | n/a | n/a | 100041.6395 |
| C7 | -49709.538 | 2.6226 | 125 | 99669.076 | 100440.738 | 100043.551 | 0.749 | 0.7966 | 0.7966 | n/a | n/a | 99678.28922 |

**Notes:**

**AHI**: Apnea Hypopnea Index

C# indicates the number of classes estimated in the model.

**LL** = *Log Likelihood*; **AIC** = *Akaike information criterion*; **BIC** = *Bayesian Information Criterion;*

**SSABIC** = *Sample Size Adjusted BIC*; **VLMR** = *Vuong-Lo-Mendell Rubin*; **LMR** = *Lo-Mendell-Rubin*; **AICc** = *Sample corrected Akaike information criterion*

* = *P* values from non-survey adjusted LCA models.

Solutions C5-C7 do not have non-survey adjusted VLMR/LMR *P* values because the models did not converge.
