# Supplementary material for: Cardiovascular correlates of sleep apnea phenotypes: Results from the Hispanic Community Health Study/Study of Latinos (HCHS/SOL)
Source: PLoS One. 2022 Apr 4;17(4):e0265151. doi: 10.1371/journal.pone.0265151 (PMC8979447; doi:10.1371/journal.pone.0265151)
Supplement: S2 Table — Models are not adjusted for survey design and subpopulated on HCHS/SOL individuals ages 45+ and with AHI ≥5. N = 3,545. (DOCX) [file pone.0265151.s004.docx]

**S2 Table. Latent Class Analysis model fit statistics. Models are not adjusted for survey design and subpopulated on HCHS/SOL individuals ages 45+ and with AHI ≥5. N=3,545.**

| **Solution** | **LL** | **Scaling Correction Factor** | **Free Parameters** | **AIC** | **BIC** | **SSABIC** | **Entropy** | **AICC** |
| --- | --- | --- | --- | --- | --- | --- | --- | --- |
| C2 | -52293.593 | 1.3747 | 35 | 104657.187 | 104873.252 | 104762.040 | 0.660 | 104657.905 |
| C3 | -51212.096 | 1.6173 | 53 | 102530.192 | 102857.376 | 102688.969 | 0.733 | 102531.832 |
| C4 | -50678.597 | 1.3956 | 71 | 101499.193 | 101937.497 | 101711.895 | 0.768 | 101502.137 |
| C5 | -50348.549 | 1.3134 | 89 | 100875.097 | 101424.52 | 101141.724 | 0.751 | 100879.734 |

**Notes:**

AHI: Apnea Hypopnea Index

C# indicates the number of classes estimated in the model.

LL = *Log Likelihood*; AIC = *Akaike information criterion*; BIC = *Bayesian Information Criterion;*

SSABIC = *Sample Size Adjusted BIC*; VLMR = *Vuong-Lo-Mendell Rubin*; LMR = *Lo-Mendell-Rubin*; AICc = *Sample corrected Akaike information criterion*
