# Supplementary material for: Cardiovascular correlates of sleep apnea phenotypes: Results from the Hispanic Community Health Study/Study of Latinos (HCHS/SOL)
Source: PLoS One. 2022 Apr 4;17(4):e0265151. doi: 10.1371/journal.pone.0265151 (PMC8979447; doi:10.1371/journal.pone.0265151)
Supplement: S3 Table — a. Symptom comparisons across sleep phenotypes relative to Asymptomatic with Mild OSA group for the primary solution. b. Symptom means and proportion contrasts across sleep phenotypes for the primary solution. (DOCX) [file pone.0265151.s005.docx]

**S3a Table. Symptom comparisons across sleep phenotypes relative to Asymptomatic with Mild OSA group for the primary solution.**

|  |  | **Insomnia OSA**  **vs**  **Asymptomatic Mild OSA** | **Symptomatic OSA**  **vs**  **Asymptomatic with Mild OSA** |
| --- | --- | --- | --- |
| **AHI*** | | -3.31*** (0.41) | 29.70*** (1.20) |
| **ESS*** | | 3.40*** (0.21) | 4.91*** (0.35) |
| **WHIIRS*** | | 8.70*** (0.18) | 5.97*** (0.27) |
| **Sleep Duration*** | | -0.20* (0.08) | -0.07 (0.09) |
| **Restless Legs†** | |  |  |
|  | No (%) | -22.82*** (2.58) | -18.88*** (3.09) |
|  | Yes (%) | 22.82*** (2.58) | 18.88*** (3.09) |
| **Naps†** | |  |  |
|  | None (%) | -11.61*** (3.11) | -12.15*** (3.37) |
|  | 1-2 (%) | 2.15 (2.39) | 0.118 (2.60) |
|  | 3-4 (%) | 5.24** (1.86) | 5.00* (2.36) |
|  | 5+ (%) | 4.22 (2.26) | 7.03** (2.44) |
| **Sleep Quality†** | |  |  |
|  | Very sound/restful (%) | -18.21*** (1.87) | -13.62*** (2.2) |
|  | Sound/restful (%) | -49.19*** (2.56) | -34.16*** (3.18) |
|  | Average quality (%) | 31.47*** (2.75) | 25.99*** (3.23) |
|  | Restless (%) | 26.94*** (1.96) | 17.19*** (1.95) |
|  | Very restless (%) | 8.98*** (0.91) | 4.6*** (1.04) |
| **Any time SpO2<90%†** | |  |  |
|  | 0% (%) | -0.73 (0.96) | -3.89*** (0.8) |
|  | >0% (%) | 0.73 (0.96) | 3.89*** (0.8) |

**Notes:**

* Differences in means relative to Asymptomatic with Mild OSA group reported with standard errors

**†**Difference in proportions relative to Asymptomatic with Mild OSAgroup reported with standard errors

*P*<0.05 (*), *P*<0.01 (**), *P*<0.001 (***)

**AHI**: Apnea-Hypopnea Index; **ESS**: Epworth Sleepiness Scale; **WHIIRS**: Women’s Health Initiative Insomnia Rating Scale; **SpO2**: Oxygen saturation

**S3b Table. Symptom means and proportion contrasts across sleep phenotypes for the primary solution.**

|  |  | **Group 1** | **Group 2** | **Group 3** |
| --- | --- | --- | --- | --- |
|  |  | **Insomnia OSA** | **Asymptomatic with Mild OSA** | **Symptomatic OSA** |
| Unweighted N's (Weighted %) | | 1596 (44.3%) | 1275 (36.2%) | 674 (19.5%) |
| **AHI*** | | 10.5 (5.2)^B,C^ | 13.8 (9.2)^A,C^ | 43.5 (23.9)^A,B^ |
| **ESS*** | | 7.3 (6.2)^B,C^ | 3.9 (3.6)^A,C^ | 8.8 (7.4)^A,B^ |
| **WHIIRS*** | | 11.2 (5.5)^B,C^ | 2.5 (2.2)^A,C^ | 8.4 (6.0)^A,B^ |
| **Sleep Duration*** | | 7.7 (1.9)^B^ | 7.9 (1.5)^A^ | 7.9 (1.6) |
| **Experience desire to move your legs because of discomfort in your legs?†** | |  |  |  |
|  | No (%) | 59.2 (2.0)^B^ | 82.1 (1.7)^A,C^ | 63.2 (2.6)^B^ |
|  | Yes (%) | 40.8 (2.0)^B^ | 17.9 (1.7)^A,C^ | 36.8 (2.6)^B^ |
| **How many 5+ minute naps do  you take in a week?†** | |  |  |  |
|  | None | 48.5 (2.1)^B^ | 60.1 (2.2)^B,C^ | 48.0 (2.7)^B^ |
|  | 1-2 | 22.3 (1.7) | 20.1 (1.7) | 20.3 (2.1) |
|  | 3-4 | 13.7 (1.6)^B^ | 8.4 (0.9)^B,C^ | 13.5 (2.2)^B^ |
|  | 5+ | 15.5 (1.8) | 11.3 (1.5)^C^ | 18.3 (1.9)^B^ |
| **How was your night's sleep  over the past 4 weeks?†** | |  |  |  |
|  | Very sound/restful | 1.1 (0.3)^B,C^ | 19.4 (1.9)^A,C^ | 5.7 (1.0)^A,B^ |
|  | Sound/restful | 9.8 (1.1)^B,C^ | 59.0 (2.3)^A,C^ | 24.8 (2.2)^A,B^ |
|  | Average quality (%) | 53.1 (2.1)^B^ | 21.7 (1.8)^A,C^ | 47.7 (2.7)^B^ |
|  | Restless (%) | 26.9 (2.0)^B,C^ | 0.0 (0.0)^A,C^ | 17.2 (1.9)^A,B^ |
|  | Very restless (%) | 9.0 (0.9)^B,C^ | 0.0 (0.0)^A,C^ | 4.6 (1.0)^A,B^ |
| **Binary Percent time SpO2<90%†** | |  |  |  |
|  | 0% | 3.3 (0.5)^C^ | 4.0 (0.8)^C^ | 0.1 (0.1)^A,B^ |
|  | >0% | 96.7 (0.5)^C^ | 96.0 (0.8)^C^ | 99.9 (0.1)^A,B^ |

**Notes**

*Group differences testing for continuous variables calculated through survey adjusted linear regression of the clustering variable on latent class membership

**†**Group differences testing for categorical variables calculated through survey adjusted proportions of the clustering variables over the latent class membership

**A:** Group differences significant at *P*<0.05 relative to the Insomnia OSA group

**B:** Group differences significant at *P*<0.05 relative to the Asymptomatic with Mild OSA group

**C:** Group differences significant at *P*<0.05 relative to the Symptomatic OSA group

**AHI**: Apnea-Hypopnea Index; **ESS**: Epworth Sleepiness Scale; **WHIIRS**: Women’s Health Initiative Insomnia Rating Scale; **SpO2**: Oxygen saturation
