# Supplementary material for: Cardiovascular correlates of sleep apnea phenotypes: Results from the Hispanic Community Health Study/Study of Latinos (HCHS/SOL)
Source: PLoS One. 2022 Apr 4;17(4):e0265151. doi: 10.1371/journal.pone.0265151 (PMC8979447; doi:10.1371/journal.pone.0265151)
Supplement: S4 Table — a. Socioeconomic and sociodemographic characteristics comparisons across sleep phenotypes relative to Asymptomatic with Mild OSA group for the primary solution. b. Socioeconomic and sociodemographic means and proportion contrasts across sleep phenotypes for the primary solution. (DOCX) [file pone.0265151.s006.docx]

**S4a Table. Socioeconomic and sociodemographic characteristics comparisons across sleep phenotypes relative to Asymptomatic with Mild OSA group for the primary solution.**

|  |  |  | **Insomnia OSA**  **vs**  **Asymptomatic with Mild OSA** | **Symptomatic OSA**  **vs**  **Asymptomatic with Mild OSA** |
| --- | --- | --- | --- | --- |
| **Sociodemographic Characteristics** | | |  |  |
|  | **Age*** | | -0.45 (0.50) | -0.36 (0.57) |
|  | **Sex†** | |  |  |
|  |  | Female (%) | 17.24*** (2.45) | -2.48 (3.08) |
|  |  | Male (%) | -17.24*** (2.45) | 2.48 (3.08) |
|  | **Race/Ethnicity†** | |  |  |
|  |  | Central American (%) | -2.04* (0.85) | -1.82 (0.98) |
|  |  | Cuban (%) | -5.08 (3.05) | 2.21 (3.03) |
|  |  | Dominican (%) | 3.39* (1.45) | 2.51 (1.74) |
|  |  | Mexican (%) | -7.69** (2.63) | -7.97** (2.76) |
|  |  | Puerto Rican (%) | 12.89*** (2.25) | 8.07*** (2.18) |
|  |  | South American (%) | -0.49 (1.03) | -1.37 (1.1) |
|  |  | Other (%) | -0.99 (0.88) | -1.63 (0.95) |
| **Socioeconomic Variables** | | |  |  |
|  | **Education†** | |  |  |
|  |  | Less than HS (%) | 5.49 (2.94) | 0.9 (3.13) |
|  |  | HS or Equivalent (%) | -2.67 (2.1) | 0.28 (2.55) |
|  |  | Greater than HS (%) | -2.82 (2.89) | -1.19 (2.99) |
|  | **Income†** | |  |  |
|  |  | <$30,000 (%) | 6.41* (2.69) | 1.52 (3.09) |
|  |  | >=$30,000 (%) | -7.02** (2.48) | -0.66 (2.94) |
|  |  | Not reported (%) | 0.61 (1.43) | -0.86 (1.81) |
|  | **Occupation Longest†** | |  |  |
|  |  | Non-skilled worker (%) | 0.47 (2.6) | -2.38 (2.81) |
|  |  | Service worker (%) | -2.71 (2.02) | -2.74 (2.45) |
|  |  | Skilled worker (%) | 4.32 (2.51) | 6.64* (2.64) |
|  |  | Professional/technical/other office worker (%) | -3.64 (2.07) | -3.72 (2.57) |
|  |  | Other (%) | 1.57 (2.46) | 2.2 (2.72) |
|  | **Occupation current + Employment status†** | |  |  |
|  |  | Non-skilled worker (%) | -3.5* (1.58) | -4.95** (1.91) |
|  |  | Service worker (%) | -0.91 (1.37) | 2.88 (1.91) |
|  |  | Skilled worker (%) | -2.31 (1.48) | 0.68 (2.34) |
|  |  | Professional/technical/other office worker (%) | -3.24** (1.23) | -3.8** (1.43) |
|  |  | Other (%) | 1.42 (1.36) | 2.08 (1.78) |
|  |  | Retired & Not employed (%) | -0.18 (2.92) | 0.55 (3.15) |
|  |  | Not retired & not employed (%) | 8.72** (2.65) | 2.55 (3.01) |
| **Acculturation** | | |  |  |
|  | **SASH Language subscale*** | | 0.22*** (0.06) | 0.21** (0.07) |
|  | **SASH Social subscale*** | | 0.12** (0.04) | 0.09* (0.04) |

**Notes:**

*Differences in means relative to Asymptomatic with Mild OSA group reported with standard errors

**†** Difference in proportions relative to Asymptomatic with Mild OSA group reported with standard errors

*P*<0.05 (*), *P*<0.01 (**), *P*<0.001 (***)

**HS**: High School; **SASH**: Short Acculturation Scale for Hispanics

**S4b Table.** Socioeconomic and sociodemographic means and proportion contrasts across sleep phenotypes for the primary solution.

|  |  |  | **Insomnia OSA** | **Asymptomatic with Mild OSA** | **Symptomatic OSA** |
| --- | --- | --- | --- | --- | --- |
| **Sociodemographic Characteristics** | | |  |  |  |
|  | **Age*** | | 58.0 (9.7) | 58.4 (9.5) | 58.0 (9.4) |
|  | **Sex†** | |  |  |  |
|  |  | Female | 56.3 (1.8)^BC^ | 39.0 (1.8)^A^ | 36.6 (2.3)^A^ |
|  |  | Male | 43.7 (1.8)^BC^ | 61.0 (1.8)^A^ | 63.4 (2.3)^A^ |
|  | **Race/Ethnicity†** | |  |  |  |
|  |  | Central American | 25.5 (2.4)^B^ | 30.6 (3.1)^A^ | 32.8 (2.8) |
|  |  | Cuban | 9.8 (1.3)^C^ | 6.4 (0.9) | 8.9 (1.6)^A^ |
|  |  | Dominican | 4.9 (0.6)^B^ | 7.0 (0.8)^A^ | 5.1 (0.8) |
|  |  | Mexican | 28.8 (1.9)^B^ | 36.5 (2.7)^AC^ | 28.5 (2.7)^B^ |
|  |  | Puerto Rican | 23.5 (2.2)^B^ | 10.6 (1.1)^AC^ | 18.7 (2.1)^B^ |
|  |  | South American | 5.1 (0.6) | 5.6 (0.8) | 4.2 (0.8) |
|  |  | Other | 2.3 (0.5) | 3.3 (0.7) | 1.6 (0.6) |
| **Socioeconomic Variables** | | |  |  |  |
|  | **Education†** | |  |  |  |
|  |  | Less than HS | 44.5 (2.0) | 39.0 (2.2) | 39.9 (2.6) |
|  |  | HS or Equivalent | 18.5 (1.3) | 21.2 (1.6) | 21.5 (2.1) |
|  |  | Greater than HS | 37.0 (2.0) | 39.8 (2.1) | 38.6 (2.5) |
|  | **Income†** | |  |  |  |
|  |  | <$30,000 | 68.3 (1.9)^B^ | 61.9 (2.3)^A^ | 63.4 (2.7) |
|  |  | >=$30,000 | 24.6 (1.7)^BC^ | 31.6 (2.2)^A^ | 30.9 (2.6)^A^ |
|  |  | Missing | 7.1 (1.0) | 6.5 (1.1) | 5.7 (1.4) |
|  | **Occupation Longest†** | |  |  |  |
|  |  | Non-skilled worker | 25.0 (1.8) | 24.5 (2.0) | 22.1 (1.9) |
|  |  | Service worker | 13.3 (1.2) | 16.0 (1.6) | 13.2 (1.8) |
|  |  | Skilled worker | 23.5 (2.0) | 19.1 (1.6)^C^ | 25.8 (2.5)^B^ |
|  |  | Professional/technical/other office worker | 15.7 (1.3) | 19.4 (1.8) | 15.7 (2.0) |
|  |  | Other | 22.5 (1.9) | 21.0 (1.7) | 23.2 (2.2) |
|  | **Occupation current + Employment status†** | | |  |  |
|  |  | Non-skilled worker | 9.5 (1.0)^B^ | 12.9 (1.3)^AC^ | 8.0 (1.4)^B^ |
|  |  | Service worker | 7.9 (1.0)^C^ | 8.8 (1.0) | 11.6 (1.6)^A^ |
|  |  | Skilled worker | 7.8 (0.8) | 10.1 (1.2) | 10.8 (2.0) |
|  |  | Professional/technical/other office worker | 4.4 (0.7)^B^ | 7.6 (1.1)^AC^ | 3.8 (0.8)^B^ |
|  |  | Other | 7.8 (1.0) | 6.3 (0.8) | 8.4 (1.5) |
|  |  | Retired & Not employed | 24.1 (2.0) | 24.3 (2.1) | 24.9 (2.4) |
|  |  | Not retired & not employed | 38.6 (2.0)^B^ | 29.9 (1.9)^A^ | 32.4 (2.4)^A^ |
| **Acculturation** | | |  |  |  |
|  | **SASH Language subscale*** | | 1.8 (1.2)^B^ | 1.6 (1.0)^A,C^ | 1.8 (1.2)^B^ |
|  | **SASH Social subscale*** | | 2.2 (0.8)^B^ | 2.1 (0.7)^A,C^ | 2.2 (0.7)^B^ |

**Notes**

*Group differences testing for continuous variables calculated through survey adjusted linear regression of the clustering variable on latent class membership

**†**Group differences testing for categorical variables calculated through survey adjusted proportions of the clustering variables over the latent class membership

**A:** Group differences significant at *P*<0.05 relative to the Insomnia group

**B:** Group differences significant at *P*<0.05 relative to the Asymptomatic with Mild OSA group

**C:** Group differences significant at *P*<0.05 relative to the Symptomatic OSA group

**HS**: High School; **SASH**: Short Acculturation Scale for Hispanics
