# Supplementary material for: Cardiovascular correlates of sleep apnea phenotypes: Results from the Hispanic Community Health Study/Study of Latinos (HCHS/SOL)
Source: PLoS One. 2022 Apr 4;17(4):e0265151. doi: 10.1371/journal.pone.0265151 (PMC8979447; doi:10.1371/journal.pone.0265151)
Supplement: S5 Table — a. Cardiovascular characteristics comparisons across sleep phenotypes relative to Asymptomatic with Mild OSA group for the primary solution. b. Cardiovascular means and proportion contrasts across sleep phenotypes for the primary solution. (DOCX) [file pone.0265151.s007.docx]

**S5a Table. Cardiovascular characteristics comparisons across sleep phenotypes relative to Asymptomatic with Mild OSA group for the primary solution.**

|  |  |  | **Insomnia vs Asymptomatic Mild OSA** | **Symptomatic OSA vs Asymptomatic Mild OSA** |  |
| --- | --- | --- | --- | --- | --- |
|  |  |  |  |  |  |
|  |  |  |  |  |  |
|  |  |  |  |  |  |
| **Cardiovascular health, risk, and disease factors** | | | |  |  |
|  | **HDL Cholesterol*** | | 1.33 (0.85) | -3.71*** (0.83) |  |
|  | **Total cholesterol*** | | -2.46 (2.50) | -3.19 (2.98) |  |
|  | **Triglycerides*** | | -9.04 (5.09) | 16.49* (7.75) |  |
|  | **BMI*** | | 0.77* (0.31) | 3.12*** (0.35) |  |
|  | **Cigarrette Usage†** | |  |  |  |
|  |  | Never | -1.3 (2.75) | -10.48** (3.26) |  |
|  |  | Former | -0.2 (2.34) | 5.24 (3.00) |  |
|  |  | Current | 1.5 (2.18) | 5.24 (2.80) |  |
|  | **Alcohol Usage†** | |  |  |  |
|  |  | Doesn't drink alcohol | 1.87 (2.73) | 3.97 (3.16) |  |
|  |  | Drinks alcohol | -1.87 (2.73) | -3.97 (3.16) |  |
|  | **CVD** | |  |  |  |
|  |  | No CVD | -14.01 (2.73)*** | -15.81 (3.06)*** |  |
|  |  | CVD | 14.01 (2.73)*** | 15.81 (3.06)*** |  |
|  | **Heart Failure†** | |  |  |  |
|  |  | No heart Failure | -1.07 (1.07) | -1.56 (1.27) |  |
|  |  | Heart Failure | 1.07 (1.07) | 1.56 (1.27) |  |
|  | **Stroke/TIA†** | |  |  |  |
|  |  | No Prevalent Stroke/TIA | -0.54 (1.44) | 1.15 (1.51) |  |
|  |  | Prevalent Stroke/TIA | 0.54 (1.44) | -1.15 (1.51) |  |
|  | **Hypertension†** | |  |  |  |
|  |  | Not hypertensive | 0.02 (2.92) | -11.91*** (3.21) |  |
|  |  | Hypertensive | -0.02 (2.92) | 11.91*** (3.21) |  |
|  | **Diabetes†** | |  |  |  |
|  |  | Non-diabetic | -0.49 (2.7) | -9.22** (3.42) |  |
|  |  | Diabetic | 0.49 (2.7) | 9.22** (3.42) |  |
|  | **FRS Score** | | -0.03*** (0.01) | 0.02 (0.01) |  |
|  | **FRS Score (3 categories)** | |  |  |  |
|  |  | <0.1 | 9.85 (2.63)*** | -6.43 (2.66)* |  |
|  |  | 0.1-<0.2 | -3.15 (2.8) | -1.15 (3.47) |  |
|  |  | >=0.2 | -6.7 (2.7)* | 7.58 (3.53)* |  |

**Notes:**

*Differences in means relative to Asymptomatic with Mild OSA group reported with standard errors

**†**Difference in proportions relative to Asymptomatic with Mild OSA group reported with standard errors

*P*<0.05 (*), *P*<0.01 (**), *P*<0.001 (***)

**CVD:** Cardiovascular disease; **HDL**: High-density lipoproteins; **BMI**: Body Mass Index; **CHD**: Coronary heart disease; **TIA**: Transient Ischemic Attack

**S5b Table. Cardiovascular means and proportion contrasts across sleep phenotypes for the primary solution.**

|  |  |  | **Asymptomatic** | **Asymptomatic with Mild Sleep Apnea** | **Symptomatic Sleep Apnea** |
| --- | --- | --- | --- | --- | --- |
|  |  |  |  |  |  |
| **Cardiovascular health, risk, and disease factors** | | | |  |  |
|  | **HDL Cholesterol*** | | 49.1 (14.7)^C^ | 47.7 (15.9)^C^ | 44.0 (12.4)^A,B^ |
|  | **Total Cholesterol*** | | 206.5 (53.1) | 209.0 (50.0) | 205.8 (55.3) |
|  | **Triglycerides*** | | 149.8 (104.6)^C^ | 158.8 (130.0)^C^ | 175.3 (157.3)^A,B^ |
|  | **BMI*** | | 31.4 (6.7)^B,C^ | 30.6 (5.9)^A,C^ | 33.7 (7.0)^A,B^ |
|  | **Cigarrette Usage†** | |  |  |  |
|  |  | Never | 54.1 (2.0)^C^ | 55.4 (1.9)^C^ | 44.9 (2.6)^AB^ |
|  |  | Former | 28.5 (1.6) | 28.7 (1.7) | 33.9 (2.6) |
|  |  | Current | 17.4 (1.6) | 15.9 (1.4) | 21.1 (2.4) |
|  | **Alcohol Usage†** | |  |  |  |
|  |  | Doesn't drink alcohol | 55.4 (1.8) | 53.6 (2.0) | 57.5 (2.4) |
|  |  | Drinks alcohol | 44.6 (1.8) | 46.4 (2.0) | 42.5 (2.4) |
|  | **CVD** | |  |  |  |
|  |  | No CVD | 49.2 (2.0)^B^ | 63.2 (1.9)^AC^ | 47.4 (2.7)^B^ |
|  |  | CVD | 50.8 (2.0)^B^ | 36.8 (1.9)^AC^ | 52.6 (2.7)^B^ |
|  | **Heart Failure†** | |  |  |  |
|  |  | No heart Failure | 96.4 (0.7) | 97.5 (0.8) | 95.9 (1.0) |
|  |  | Heart Failure | 3.6 (0.7) | 2.5 (0.8) | 4.1 (1.0) |
|  | **Stroke/TIA†** | |  |  |  |
|  |  | No Prevalent Stroke/TIA | 94.1 (0.8) | 94.6 (1.1) | 95.8 (0.9) |
|  |  | Prevalent Stroke/TIA | 5.9 (0.8) | 5.4 (1.1) | 4.2 (0.9) |
|  | **Hypertension†** | |  |  |  |
|  |  | Not hypertensive | 43.7 (2.0)^C^ | 43.7 (2.1)^C^ | 31.8 (2.4)^AB^ |
|  |  | Hypertensive | 56.3 (2.0)^C^ | 56.3 (2.1)^C^ | 68.2 (2.4)^AB^ |
|  | **Diabetes†** | |  |  |  |
|  |  | Non-diabetic | 66.1 (1.9)^C^ | 66.6 (2.0)^C^ | 57.3 (2.7)^AB^ |
|  |  | Diabetic | 33.9 (1.9)^C^ | 33.4 (2.0)^C^ | 42.7 (2.7)^AB^ |
|  | **FRS Score** | | 0.14 (0.13)^BC^ | 0.15 (0.13)^A^ | 0.20 (0.15)^A^ |
|  | **FRS Score (3 categories)** | |  |  |  |
|  |  | <0.1 | 37.2 (2.0)^BC^ | 27.4 (1.7)^AC^ | 21.0 (1.9)^AB^ |
|  |  | 0.1-<0.2 | 32.3 (2.0) | 35.5 (2.0) | 34.3 (2.6) |
|  |  | >=0.2 | 30.4 (1.9)^BC^ | 37.1 (2.2)^AC^ | 44.7 (2.8)^AB^ |

**Notes**

*Group differences testing for continuous variables calculated through survey adjusted linear regression of the clustering variable on latent class membership

**†**Group differences testing for categorical variables calculated through survey adjusted proportions of the clustering variables over the latent class membership

**A:** Group differences significant at *P*<0.05 relative to the Insomnia group

**B:** Group differences significant at *P*<0.05 relative to the Asymptomatic with Mild OSA group

**C:** Group differences significant at *P*<0.05 relative to the Symptomatic OSA group

**CVD:** Cardiovascular disease; **HDL**: High-density lipoproteins; **BMI**: Body Mass Index; **CHD**: Coronary heart disease; **TIA**: Transient Ischemic Attack
