# Supplementary material for: Cardiovascular correlates of sleep apnea phenotypes: Results from the Hispanic Community Health Study/Study of Latinos (HCHS/SOL)
Source: PLoS One. 2022 Apr 4;17(4):e0265151. doi: 10.1371/journal.pone.0265151 (PMC8979447; doi:10.1371/journal.pone.0265151)
Supplement: S6 Table — Unweighted N = 5,588. (DOCX) [file pone.0265151.s008.docx]

**S6 Table. Sociodemographic, socioeconomic, and cardiovascular characteristics of HCHS/SOL individuals for three-group solution and AHI<5 individuals with survey design adjustment and subpopulation on Ages 45+. Unweighted N=5,588.**

|  |  | **AHI < 5**  **n=3308** | **Asymptomatic Mild OSA**  **n=816** | **Insomnia**  **n=1,047** | **Symptomatic OSA**  **n=417** | **Total** | **p-value** |
| --- | --- | --- | --- | --- | --- | --- | --- |
| **Age at V1*** | | 55.09 (8.12) | 58.15 (7.84) | 58.18 (7.81) | 57.09 (7.48) | 56.39 (8.11) | p<0.001 |
| **Sex‡** | |  |  |  |  |  |  |
|  | Female | 61.26 (1.32) | 39.95 (2.28) | 55.99 (2.02) | 38.14 (2.86) | 54.84 (0.92) | p<0.001 |
|  | Male | 38.74 (1.32) | 60.05 (2.28) | 44.01 (2.02) | 61.86 (2.86) | 45.16 (0.92) |  |
| **Latino Background‡** | |  |  |  |  |  |  |
|  | Dominican | 10.93 (1.02) | 6.36 (1.06) | 9.97 (1.45) | 8.72 (1.78) | 9.82 (0.78) | p<0.001 |
|  | Central American | 8.02 (0.73) | 8.56 (1.27) | 5.60 (0.78) | 6.25 (1.12) | 7.47 (0.58) |  |
|  | Cuban | 21.21 (2.00) | 32.05 (3.70) | 25.04 (2.80) | 31.00 (3.55) | 24.54 (2.00) |  |
|  | Mexican | 38.61 (2.05) | 37.44 (3.03) | 33.73 (2.60) | 33.50 (3.33) | 37.02 (1.83) |  |
|  | Puerto Rican | 15.92 (1.06) | 9.07 (1.22) | 21.03 (1.73) | 15.88 (2.41) | 15.83 (0.89) |  |
|  | South American | 5.31 (0.52) | 6.53 (1.24) | 4.63 (0.72) | 4.65 (1.04) | 5.32 (0.41) |  |
| **Cigarette Usage‡** | |  |  |  |  |  |  |
|  | Never | 57.46 (1.36) | 60.51 (2.36) | 53.11 (2.29) | 47.37 (3.24) | 56.25 (1.03) | p<0.001 |
|  | Former | 23.08 (1.13) | 25.30 (2.05) | 31.64 (2.23) | 34.37 (3.32) | 26.09 (0.95) |  |
|  | Current | 19.45 (1.07) | 14.20 (1.75) | 15.25 (1.56) | 18.26 (2.42) | 17.66 (0.80) |  |
| **Alcohol Usage‡** | |  |  |  |  |  |  |
|  | Doesn't drink alcohol | 54.52 (1.40) | 53.36 (2.47) | 52.75 (2.15) | 54.32 (3.26) | 53.96 (1.03) | 0.899 |
|  | Drinks alcohol | 45.48 (1.40) | 46.64 (2.47) | 47.25 (2.15) | 45.68 (3.26) | 46.04 (1.03) |  |
| **BMI at V1*** | | 28.32 (4.98) | 30.50 (4.72) | 31.04 (5.24) | 33.64 (5.76) | 29.75 (5.42) | p<0.001 |
| **HDL at V1*** | | 51.27 (13.98) | 48.61 (13.00) | 48.44 (11.64) | 44.08 (9.96) | 49.62 (13.16) | p<0.001 |
| **Total Cholesterol at V1*** | | 210.61 (46.80) | 211.09 (41.32) | 205.47 (42.79) | 206.84 (45.06) | 209.19 (44.43) | 0.078 |
| **Triglycerides at V1*** | | 146.83 (179.20) | 154.91 (98.72) | 150.78 (87.77) | 178.02 (154.93) | 151.17 (140.62) | 0.008 |
| **AHI*** | | 1.68 (1.48) | 13.92 (7.51) | 10.43 (4.10) | 41.45 (19.31) | 8.74 (13.08) | p<0.001 |
| **ESS*** | | 5.49 (4.87) | 3.86 (2.95) | 7.36 (5.07) | 9.31 (5.92) | 6.04 (4.98) | p<0.001 |
|  |  |  |  |  |  |  |  |
| **Cardiovascular Disease** | |  |  |  |  |  |  |
| **Prevalent CVD event** | |  |  |  |  |  |  |
|  | No CVD event | 60.69 (1.24) | 63.02 (2.44) | 49.70 (2.32) | 48.35 (3.44) | 57.84 (1.00) | p<0.001 |
|  | CVD event | 39.31 (1.24) | 36.98 (2.44) | 50.30 (2.32) | 51.65 (3.44) | 42.16 (1.00) |  |
| **Heart failure** | |  |  |  |  |  |  |
|  | No heart Failure | 97.62 (0.38) | 98.27 (0.72) | 97.80 (0.63) | 95.88 (1.32) | 97.62 (0.31) | 0.295 |
|  | Heart Failure | 2.38 (0.38) | 1.73 (0.72) | 2.20 (0.63) | 4.12 (1.32) | 2.38 (0.31) |  |
| **Stroke‡** | |  |  |  |  |  |  |
|  | No Prevalent Stroke/TIA | 97.49 (0.42) | 95.39 (1.59) | 94.32 (1.09) | 96.94 (0.87) | 96.47 (0.42) | 0.027 |
|  | Prevalent Stroke/TIA | 2.51 (0.42) | 4.61 (1.59) | 5.68 (1.09) | 3.06 (0.87) | 3.53 (0.42) |  |
| **Diabetes‡** | |  |  |  |  |  |  |
|  | Non-diabetic | 76.49 (1.06) | 67.84 (2.49) | 64.99 (2.49) | 60.90 (3.12) | 71.49 (0.94) | p<0.001 |
|  | Diabetic | 23.51 (1.06) | 32.16 (2.49) | 35.01 (2.49) | 39.10 (3.12) | 28.51 (0.94) |  |
| **Hypertension‡** | |  |  |  |  |  |  |
|  | Not hypertensive | 61.71 (1.26) | 45.83 (2.69) | 45.47 (2.48) | 34.43 (2.93) | 53.62 (1.06) | p<0.001 |
|  | Hypertensive | 38.29 (1.26) | 54.17 (2.69) | 54.53 (2.48) | 65.57 (2.93) | 46.38 (1.06) |  |
| **FRS** | | 0.13 (0.12) | 0.19 (0.14) | 0.17 (0.13) | 0.20 (0.13) | 0.16 (0.13) | p<0.001 |
| **FRS (3 Categories)** | |  |  |  |  |  |  |
|  | <0.1 | 51.49 (1.34) | 29.44 (2.09) | 36.29 (2.42) | 24.24 (2.73) | 42.63 (0.97) | p<0.001 |
|  | 0.1-<0.2 | 27.83 (1.29) | 36.69 (2.46) | 32.91 (2.10) | 36.42 (3.26) | 30.99 (0.96) |  |
|  | >=0.2 | 20.68 (1.26) | 33.86 (2.84) | 30.81 (2.64) | 39.35 (3.39) | 26.38 (1.09) |  |

**Notes:**

* Means and Standard Deviations are presented; **†** % and Standard Errors (SEs) are presented

*P* value: Pearson's chi square test for continuous variables; Regression based F test for categorical variables

**BMI:** body mass index; **HDL**: high-density lipoprotein; **AHI**: Apnea-Hypopnea Index; **ESS**: Epworth Sleepiness Scale; **CVD:** cardiovascular disease; **TIA**: **FRS:** Framingham Risk Score
