# Supplementary material for: Cardiovascular correlates of sleep apnea phenotypes: Results from the Hispanic Community Health Study/Study of Latinos (HCHS/SOL)
Source: PLoS One. 2022 Apr 4;17(4):e0265151. doi: 10.1371/journal.pone.0265151 (PMC8979447; doi:10.1371/journal.pone.0265151)
Supplement: S7 Table — (DOCX) [file pone.0265151.s009.docx]

**S7 Table. Associations between OSA phenotypes and incidence of cardiovascular risks.**

|  |  | **Prevalence at V1** | | | **Prevalence at V2** | | | **Incidence** | | |
| --- | --- | --- | --- | --- | --- | --- | --- | --- | --- | --- |
|  |  | **Crude** | **Demographic Adjusted** | **Full Adjusted** | **Crude** | **Demographic Adjusted** | **Full Adjusted** | **Crude** | **Demographic Adjusted** | **Full Adjusted** |
|  |  | **RRR / 95% CI /**  **p-value** | **RRR / 95% CI /**  **p-value** | **RRR / 95% CI /**  **p-value** | **RRR / 95% CI /**  **p-value** | **RRR / 95% CI /**  **p-value** | **RRR / 95% CI /**  **p-value** | **IRR / 95% CI /**  **p-value** | **IRR / 95% CI /**  **p-value** | **IRR / 95% CI /**  **p-value** |
| **CVD events** | |  |  |  |  |  |  |  |  |  |
|  | AHI<5 | ref | ref | ref | ref | ref | ref | ref | ref | ref |
|  |  |  |  |  |  |  |  |  |  |  |
|  | Asymptomatic | 0.76 [0.55;1.05] | 0.76 [0.54;1.08] | 0.68* [0.49;0.96] | 2.21*** [1.58;3.10] | 1.14 [0.76;1.72] | 1.09 [0.69;1.71] | 1.52*** [1.29;1.79] | 1.09 [0.93;1.28] | 1.09 [0.93;1.27] |
|  |  | p=0.099 | p=0.124 | p=0.030 | p<0.001 | p=0.535 | p=0.712 | p<0.001 | p=0.297 | p=0.269 |
|  | Disturbed sleep | 1.23 [1.00;1.52] | 1.20 [0.96;1.49] | 1.02 [0.82;1.26] | 2.44*** [1.98;3.02] | 1.53*** [1.19;1.97] | 1.34* [1.01;1.76] | 1.60*** [1.43;1.78] | 1.23*** [1.09;1.39] | 1.16* [1.02;1.32] |
|  |  | p=0.051 | p=0.106 | p=0.872 | p<0.001 | p=0.001 | p=0.039 | p<0.001 | p=0.001 | p=0.019 |
|  | Insomnia | 1.93*** [1.53;2.44] | 1.70*** [1.33;2.18] | 1.42** [1.10;1.84] | 1.75*** [1.38;2.23] | 1.38* [1.02;1.85] | 1.08 [0.79;1.47] | 1.31** [1.10;1.57] | 1.16 [0.99;1.36] | 1.05 [0.89;1.24] |
|  |  | p<0.001 | p<0.001 | p=0.007 | p<0.001 | p=0.035 | p=0.619 | p=0.003 | p=0.068 | p=0.557 |
| **Heart failure** | |  |  |  |  |  |  |  |  |  |
|  | AHI<5 | ref | ref | ref | ref | ref | ref | ref | ref | ref |
|  |  |  |  |  |  |  |  |  |  |  |
|  | Asymptomatic | 0.65 [0.14;3.15] | 0.58 [0.13;2.53] | 0.54 [0.13;2.23] | 0.95 [0.35;2.58] | 0.80 [0.30;2.10] | 0.75 [0.29;1.89] | 1.76 [0.47;6.65] | 1.41 [0.36;5.46] | 1.37 [0.37;5.11] |
|  |  | p=0.595 | p=0.469 | p=0.392 | p=0.917 | p=0.649 | p=0.535 | p=0.402 | p=0.618 | p=0.635 |
|  | Disturbed sleep | 0.89 [0.49;1.60] | 0.77 [0.43;1.39] | 0.62 [0.32;1.19] | 1.40 [0.87;2.25] | 1.15 [0.72;1.84] | 0.98 [0.58;1.66] | 2.68** [1.38;5.20] | 2.04* [1.11;3.77] | 1.89 [0.96;3.73] |
|  |  | p=0.687 | p=0.388 | p=0.149 | p=0.166 | p=0.547 | p=0.937 | p=0.004 | p=0.023 | p=0.067 |
|  | Insomnia | 1.47 [0.81;2.67] | 1.29 [0.71;2.35] | 1.05 [0.52;2.10] | 2.51*** [1.53;4.10] | 2.21** [1.36;3.59] | 1.88* [1.08;3.28] | 4.98*** [2.48;9.98] | 4.35*** [2.26;8.37] | 4.01*** [1.91;8.41] |
|  |  | p=0.201 | p=0.406 | p=0.894 | p<0.001 | p=0.001 | p=0.026 | p<0.001 | p<0.001 | p<0.001 |
| **Stroke** | |  |  |  |  |  |  |  |  |  |
|  | AHI<5 | ref | ref | ref | ref | ref | ref | ref | ref | ref |
|  |  |  |  |  |  |  |  |  |  |  |
|  | Asymptomatic | 2.11 [0.98;4.57] | 1.81 [0.86;3.79] | 1.63 [0.73;3.65] | 2.15* [1.16;3.99] | 1.88* [1.02;3.43] | 1.71 [0.89;3.28] | 2.21 [0.81;6.02] | 1.96 [0.71;5.42] | 1.85 [0.65;5.30] |
|  |  | p=0.058 | p=0.117 | p=0.235 | p=0.015 | p=0.041 | p=0.107 | p=0.121 | p=0.195 | p=0.249 |
|  | Disturbed sleep | 1.48 [0.74;2.96] | 1.28 [0.69;2.36] | 1.07 [0.59;1.93] | 1.70* [1.02;2.83] | 1.47 [0.92;2.33] | 1.28 [0.81;2.03] | 2.08* [1.09;3.99] | 1.79 [0.92;3.48] | 1.75 [0.90;3.42] |
|  |  | p=0.263 | p=0.431 | p=0.830 | p=0.041 | p=0.106 | p=0.283 | p=0.027 | p=0.086 | p=0.099 |
|  | Insomnia | 2.76*** [1.61;4.75] | 2.12** [1.23;3.66] | 1.75 [0.96;3.21] | 2.31*** [1.46;3.66] | 1.87** [1.17;2.97] | 1.61 [0.98;2.65] | 1.35 [0.62;2.94] | 1.18 [0.54;2.59] | 1.13 [0.50;2.52] |
|  |  | p<0.001 | p=0.007 | p=0.069 | p<0.001 | p=0.009 | p=0.060 | p=0.447 | p=0.672 | p=0.771 |
| **Diabetes** | |  |  |  |  |  |  |  |  |  |
|  | AHI<5 | ref | ref | ref | ref | ref | ref | ref | ref | ref |
|  |  |  |  |  |  |  |  |  |  |  |
|  | Asymptomatic | 1.28 [0.94;1.73] | 1.19 [0.87;1.62] | 0.96 [0.69;1.33] | 1.58** [1.17;2.13] | 1.49* [1.08;2.03] | 1.17 [0.83;1.65] | 1.90** [1.26;2.84] | 1.84** [1.22;2.77] | 1.48 [0.98;2.24] |
|  |  | p=0.116 | p=0.286 | p=0.816 | p=0.003 | p=0.014 | p=0.364 | p=0.002 | p=0.004 | p=0.066 |
|  | Disturbed sleep | 1.98*** [1.55;2.52] | 1.79*** [1.41;2.27] | 1.37* [1.07;1.75] | 2.29*** [1.85;2.83] | 2.08*** [1.69;2.57] | 1.54*** [1.23;1.92] | 2.07*** [1.63;2.62] | 1.99*** [1.58;2.51] | 1.53*** [1.20;1.94] |
|  |  | p<0.001 | p<0.001 | p=0.013 | p<0.001 | p<0.001 | p<0.001 | p<0.001 | p<0.001 | p=0.001 |
|  | Insomnia | 1.67*** [1.31;2.12] | 1.48** [1.15;1.89] | 1.06 [0.80;1.40] | 1.91*** [1.52;2.39] | 1.72*** [1.37;2.15] | 1.17 [0.92;1.50] | 1.89*** [1.38;2.59] | 1.77*** [1.31;2.40] | 1.28 [0.94;1.76] |
|  |  | p<0.001 | p=0.002 | p=0.675 | p<0.001 | p<0.001 | p=0.202 | p<0.001 | p<0.001 | p=0.116 |
| **Hypertension** | |  |  |  |  |  |  |  |  |  |
|  | AHI<5 | ref | ref | ref | ref | ref | ref | ref | ref | ref |
|  |  |  |  |  |  |  |  |  |  |  |
|  | Asymptomatic | 2.08*** [1.58;2.74] | 1.85*** [1.33;2.57] | 1.46* [1.04;2.05] | 1.98*** [1.45;2.70] | 1.79*** [1.33;2.41] | 1.44* [1.06;1.95] | 1.58** [1.19;2.10] | 1.52** [1.18;1.97] | 1.40* [1.07;1.82] |
|  |  | p<0.001 | p<0.001 | p=0.031 | p<0.001 | p<0.001 | p=0.020 | p=0.002 | p=0.001 | p=0.013 |
|  | Disturbed sleep | 1.84*** [1.51;2.24] | 1.60*** [1.30;1.96] | 1.22 [0.98;1.51] | 2.00*** [1.63;2.44] | 1.82*** [1.47;2.24] | 1.41** [1.13;1.76] | 1.44*** [1.16;1.79] | 1.41** [1.14;1.74] | 1.27* [1.02;1.58] |
|  |  | p<0.001 | p<0.001 | p=0.075 | p<0.001 | p<0.001 | p=0.002 | p=0.001 | p=0.002 | p=0.033 |
|  | Insomnia | 2.68*** [2.12;3.39] | 2.23*** [1.75;2.83] | 1.63*** [1.26;2.11] | 2.16*** [1.71;2.73] | 1.77*** [1.36;2.31] | 1.30 [0.99;1.71] | 1.62*** [1.30;2.02] | 1.51*** [1.20;1.89] | 1.32* [1.05;1.67] |
|  |  | p<0.001 | p<0.001 | p<0.001 | p<0.001 | p<0.001 | p=0.061 | p<0.001 | p<0.001 | p=0.019 |
| **FRS 3 categories** | |  |  |  |  |  |  |  |  |  |
|  | **FRS < 0.1** |  |  |  |  |  |  |  |  |  |
|  | AHI<5 | ref | ref | ref | ref | ref | ref | ref | ref | ref |
|  |  |  |  |  |  |  |  |  |  |  |
|  | Asymptomatic | ref | ref | ref | ref | ref | ref | ref | ref | ref |
|  |  |  |  |  |  |  |  |  |  |  |
|  | Disturbed sleep | ref | ref | ref | ref | ref | ref | ref | ref | ref |
|  |  |  |  |  |  |  |  |  |  |  |
|  | Insomnia | ref | ref | ref | ref | ref | ref | ref | ref | ref |
|  |  |  |  |  |  |  |  |  |  |  |
|  |  |  |  |  |  |  |  |  |  |  |
|  | **0.1 <= FRS < 0.2** |  |  |  |  |  |  |  |  |  |
|  | AHI<5 | ref | ref | ref | ref | ref | ref | ref | ref | ref |
|  |  |  |  |  |  |  |  |  |  |  |
|  | Asymptomatic | 2.19*** [1.52;3.17] | 1.23 [0.86;1.78] | 1.10 [0.72;1.68] | 1.93** [1.28;2.92] | 1.17 [0.78;1.77] | 1.01 [0.66;1.55] | 1.53 [0.93;2.51] | 1.17 [0.71;1.93] | 0.90 [0.56;1.46] |
|  |  | p<0.001 | p=0.260 | p=0.651 | p=0.002 | p=0.446 | p=0.967 | p=0.093 | p=0.530 | p=0.677 |
|  | Disturbed sleep | 2.06*** [1.65;2.57] | 1.39* [1.08;1.78] | 1.21 [0.90;1.63] | 1.91*** [1.48;2.47] | 1.41* [1.06;1.88] | 1.17 [0.86;1.59] | 1.77** [1.25;2.49] | 1.48* [1.04;2.12] | 1.18 [0.81;1.70] |
|  |  | p<0.001 | p=0.010 | p=0.201 | p<0.001 | p=0.017 | p=0.318 | p=0.001 | p=0.031 | p=0.391 |
|  | Insomnia | 1.95*** [1.49;2.54] | 1.60** [1.21;2.14] | 1.36 [0.98;1.88] | 1.63*** [1.25;2.15] | 1.35 [0.99;1.82] | 0.97 [0.69;1.35] | 1.48* [1.04;2.12] | 1.30 [0.89;1.91] | 0.91 [0.59;1.40] |
|  |  | p<0.001 | p=0.001 | p=0.064 | p<0.001 | p=0.055 | p=0.841 | p=0.030 | p=0.179 | p=0.674 |
|  |  |  |  |  |  |  |  |  |  |  |
|  | **FRS>=0.2** |  |  |  |  |  |  |  |  |  |
|  | AHI<5 | ref | ref | ref | ref | ref | ref | ref | ref | ref |
|  |  |  |  |  |  |  |  |  |  |  |
|  | Asymptomatic | 2.70*** [1.86;3.91] | 1.03 [0.65;1.64] | 0.99 [0.59;1.67] | 3.17*** [2.12;4.75] | 1.33 [0.83;2.13] | 1.17 [0.66;2.07] | 2.23 [0.87;5.71] | 2.05 [0.80;5.26] | 1.41 [0.53;3.77] |
|  |  | p<0.001 | p=0.886 | p=0.980 | p<0.001 | p=0.239 | p=0.579 | p=0.094 | p=0.133 | p=0.488 |
|  | Disturbed sleep | 2.84*** [2.12;3.82] | 1.40* [1.01;1.94] | 1.14 [0.76;1.71] | 3.58*** [2.76;4.65] | 2.12*** [1.54;2.91] | 1.68** [1.17;2.41] | 3.80*** [2.22;6.49] | 3.05*** [1.70;5.48] | 2.30** [1.28;4.16] |
|  |  | p<0.001 | p=0.044 | p=0.521 | p<0.001 | p<0.001 | p=0.005 | p<0.001 | p<0.001 | p=0.006 |
|  | Insomnia | 2.34*** [1.72;3.17] | 1.85** [1.22;2.81] | 1.50 [0.91;2.49] | 2.37*** [1.79;3.16] | 1.83** [1.26;2.64] | 1.19 [0.80;1.77] | 1.91* [1.01;3.59] | 1.59 [0.84;3.01] | 1.02 [0.52;2.00] |
|  |  | p<0.001 | p=0.004 | p=0.115 | p<0.001 | p=0.001 | p=0.396 | p=0.045 | p=0.152 | p=0.947 |

**Notes:**

**Crude**: Exposure**; Demographic Adjusted**: M1 + Age, Sex, Latino Background; **Full Adjusted**: M2 + BMI, cigarette usage, alcohol usage, HDL cholesterol, total cholesterol, and trigylcerides.

**RRR**: relative risk ratio; **IRR**: incidence rate ratio; **AHI**: apnea-hypopnea index

* = p<0.05; ** = p<0.01; *** = p<0.001
