# Supplementary material for: Cardiovascular correlates of sleep apnea phenotypes: Results from the Hispanic Community Health Study/Study of Latinos (HCHS/SOL)
Source: PLoS One. 2022 Apr 4;17(4):e0265151. doi: 10.1371/journal.pone.0265151 (PMC8979447; doi:10.1371/journal.pone.0265151)
Supplement: S8 Table — Models are adjusted for survey design and subpopulated on HCHS/SOL individuals ages 45+. a. Latent class analysis model fit statistics. Models are not adjusted for survey design and subpopulated on HCHS/SOL individuals ages 45+. Unweighted N = 9,617. (DOCX) [file pone.0265151.s010.docx]

**S8 Table.** **Latent Class Analysis model fit statistics. Models are adjusted for survey design and subpopulated on HCHS/SOL individuals ages 45+. Unweighted N=9,617.**

| **Solution** | **LL** | **Scaling Correction Factor** | **Free Parameters** | **AIC** | **BIC** | **SSABIC** | **Entropy** | **VLMR *P* value** | **LMR *P* value** | **VLMR *P* value*** | **LMR *P* value*** | **AICc** |
| --- | --- | --- | --- | --- | --- | --- | --- | --- | --- | --- | --- | --- |
| C2 | -137371 | 3.5200 | 35 | 274811.3 | 275062.3 | 274951.1 | 0.675 | *P*<0.0000 | *P*<0.0000 | *P*<0.0000 | *P*<0.0000 | 274811.6 |
| C3 | -134662 | 7.9016 | 53 | 269429.7 | 269809.8 | 269641.4 | 0.725 | 0.7429 | 0.7431 | 0.0004 | 0.0004 | 269430.3 |
| C4 | -132783 | 3.8571 | 71 | 265708.8 | 266217.9 | 265992.3 | 0.726 | 0.2396 | 0.2396 | P<0.0000 | P<0.0000 | 265709.8 |
| C5 | -131559 | 3.0215 | 89 | 263296.8 | 263935.1 | 263652.2 | 0.754 | 0.4002 | 0.4011 | P<0.0000 | P<0.0000 | 263298.5 |
| C6 | -130732 | 3.7312 | 107 | 261679.0 | 262446.3 | 262106.3 | 0.756 | 0.7353 | 0.7359 | 0.4364 | 0.4385 | 261681.4 |
| C7 | -130171 | 4.0475 | 125 | 260591.5 | 261487.9 | 261090.7 | 0.739 | 0.7911 | 0.7912 | n/a | n/a | 260594.8 |

**Notes:**

C# indicates the number of classes estimated in the model.

**LL** = *Log Likelihood*; **AIC** = *Akaike information criterion*; **BIC** = *Bayesian Information Criterion;*

**SSABIC** = *Sample Size Adjusted BIC*; **VLMR** = *Vuong-Lo-Mendell Rubin*; **LMR** = *Lo-Mendell-Rubin*; **AICc** = *Sample corrected Akaike information criterion*

* = *P*-values from non-survey adjusted LCA

Solutions C5-C7 do not have non-survey adjusted VLMR/LMR p-values because the models did not converge.

**S8a Table.** Latent Class Analysis model fit statistics. Models are not adjusted for survey design and subpopulated on HCHS/SOL individuals ages 45+. Unweighted N=9,617.

| **Solution** | **LL** | **Scaling Correction Factor** | **Free Parameters** | **AIC** | **BIC** | **SSABIC** | **Entropy** | **AICc** |
| --- | --- | --- | --- | --- | --- | --- | --- | --- |
| C2 | -137436.0 | 1.3675 | 35 | 274941.98 | 275192.98 | 275081.75 | 0.688 | 274942 |
| C3 | -134700.9 | 1.6503 | 53 | 269507.77 | 269887.85 | 269719.43 | 0.733 | 269508 |
| C4 | -132896.4 | 1.5532 | 71 | 265934.75 | 266443.91 | 266218.28 | 0.723 | 265936 |
| C5 | -131830.1 | 1.5050 | 89 | 263838.17 | 264476.41 | 264193.59 | 0.752 | 263840 |
| C6 | -131244.6 | 1.6896 | 107 | 262703.29 | 263470.61 | 263130.58 | 0.745 | 262706 |

**Notes:**

C# indicates the number of classes estimated in the model.

LL = *Log Likelihood*; AIC = *Akaike information criterion*; BIC = *Bayesian Information Criterion;*

SSABIC = *Sample Size Adjusted BIC*; VLMR = *Vuong-Lo-Mendell Rubin*; LMR = *Lo-Mendell-Rubin*; AICc = *Sample corrected Akaike information criterion*
