# Supplementary material for: Cardiovascular correlates of sleep apnea phenotypes: Results from the Hispanic Community Health Study/Study of Latinos (HCHS/SOL)
Source: PLoS One. 2022 Apr 4;17(4):e0265151. doi: 10.1371/journal.pone.0265151 (PMC8979447; doi:10.1371/journal.pone.0265151)
Supplement: S9 Table — Unweighted N = 9,617. (DOCX) [file pone.0265151.s011.docx]

**S9 Table. Symptom summary of HCHS/SOL individuals for four-group solution with survey design adjustment and subpopulation on ages 45+ (supplementary solution). Unweighted N=9,617.**

|  |  | **Insomnia** | **Asymptomatic with mild OSA** | **Symptomatic OSA** | **Asymptomatic** | **Total** | ***P* value** |
| --- | --- | --- | --- | --- | --- | --- | --- |
| Unweighted n (Weighted %) | | 2523 (25.9%) | 2443 (25.3%) | 1691 (19.0%) | 2960 (29.8%) |  |  |
| **AHI*** | | 4.6 (4.7) | 7.5 (6.1) | 28.6 (23.5) | 1.0 (1.1) | 8.9 (16.8) | *P*<0.001 |
| **ESS*** | | 6.7 (6.5) | 3.4 (3.2) | 9.1 (7.0) | 5.5 (5.4) | 6.0 (6.1) | *P*<0.001 |
| **WHIIRS*** | | 14.2 (4.6) | 2.9 (2.7) | 7.6 (5.7) | 6.4 (4.9) | 7.8 (6.8) | *P*<0.001 |
| **Sleep Duration*** | | 7.8 (2.1) | 8.0 (1.5) | 7.8 (1.7) | 7.9 (1.6) | 7.9 (1.7) | *P*=0.003 |
| **Restless Legs†** | |  |  |  |  |  |  |
|  | No | 50.8 (1.7) | 82.4 (1.1) | 66.6 (1.7) | 78.4 (1.1) | 70.0 (0.8) | *P*<0.001 |
|  | Yes | 49.2 (1.7) | 17.6 (1.1) | 33.4 (1.7) | 21.6 (1.1) | 30.0 (0.8) |  |
| **Naps per week†** | |  |  |  |  |  |  |
|  | None | 56.8 (1.8) | 61.5 (1.5) | 43.6 (1.9) | 55.3 (1.6) | 55.1 (1.0) | *P*<0.001 |
|  | 1-2 | 18.2 (1.1) | 18.3 (1.1) | 21.5 (1.4) | 23.1 (1.3) | 20.3 (0.7) |  |
|  | 3-4 | 11.1 (1.2) | 9.0 (0.7) | 12.9 (1.1) | 9.6 (0.8) | 10.5 (0.5) |  |
|  | 5+ | 13.9 (1.1) | 11.1 (1.2) | 22.0 (1.6) | 11.9 (0.9) | 14.2 (0.6) |  |
| **Sleep Quality†** | |  |  |  |  |  |  |
|  | Very sound/restful | 0.0 (0.0) | 17.0 (1.2) | 8.2 (0.9) | 12.6 (1.2) | 9.6 (0.5) | *P*<0.001 |
|  | Sound/restful | 0.8 (0.2) | 54.5 (1.6) | 31.1 (1.8) | 31.0 (1.6) | 29.2 (0.8) |  |
|  | Average | 31.7 (1.4) | 28.5 (1.3) | 45.4 (1.9) | 51.3 (1.5) | 39.3 (0.8) |  |
|  | Restless | 48.8 (1.6) | 0.0 (0.0) | 12.9 (1.2) | 4.8 (0.6) | 16.5 (0.6) |  |
|  | Very restless | 18.7 (1.4) | 0.0 (0.0) | 2.4 (0.5) | 0.3 (0.2) | 5.4 (0.4) |  |
| **Any time SpO2<90%†** | |  |  |  |  |  |  |
|  | No | 19.4 (1.2) | 5.0 (0.6) | 0.2 (0.1) | 62.8 (1.7) | 25.3 (0.7) | *P*<0.001 |
|  | Yes | 80.6 (1.2) | 95.0 (0.6) | 99.8 (0.1) | 37.2 (1.7) | 74.7 (0.7) |  |

**Notes:**

* Means and Standard Deviations are presented **; †** % and Standard Errors (SEs) are presented

*P* value: Pearson's chi square test for continuous variables; Regression based F test for categorical variables

**AHI**: Apnea-Hypopnea Index; **ESS**: Epworth Sleepiness Scale; **WHIIRS**: Women’s Health Initiative Insomnia Rating Scale; **SpO2**: Oxygen saturation
