# Supplementary material for: Cardiovascular correlates of sleep apnea phenotypes: Results from the Hispanic Community Health Study/Study of Latinos (HCHS/SOL)
Source: PLoS One. 2022 Apr 4;17(4):e0265151. doi: 10.1371/journal.pone.0265151 (PMC8979447; doi:10.1371/journal.pone.0265151)
Supplement: S10 Table — (DOCX) [file pone.0265151.s012.docx]

**S10 Table. Baseline sociodemographic, socioeconomics, and cardiovascular characteristics of HCHS/SOL individuals by supplementary solution derived sleep phenotypes.**

|  |  | **Insomnia** | **Asymptomatic with Mild OSA** | **Symptomatic OSA** | **Asymptomatic** | **Total** | **p-value** |
| --- | --- | --- | --- | --- | --- | --- | --- |
| **Age (years)*** | | 56.2 (9.5) | 57.1 (10.0) | 58.4 (9.6) | 54.8 (9.9) | 56.5 (9.9) | P<0.001 |
| **Sex†** | |  |  |  |  |  |  |
|  | Female | 63.1 (1.6) | 46.6 (1.3) | 41.3 (1.7) | 62.5 (1.4) | 54.6 (0.7) | P<0.001 |
|  | Male | 36.9 (1.6) | 53.4 (1.3) | 58.7 (1.7) | 37.5 (1.4) | 45.4 (0.7) |  |
| **Race/Ethnicity†** | |  |  |  |  |  |  |
|  | Cuban | 28.4 (2.7) | 31.6 (2.5) | 28.5 (2.3) | 21.3 (2.0) | 27.1 (2.1) | P<0.001 |
|  | Central American | 6.4 (0.6) | 6.8 (0.7) | 5.1 (0.6) | 7.2 (0.6) | 6.5 (0.4) |  |
|  | Dominican | 10.5 (1.0) | 7.8 (0.8) | 9.2 (1.3) | 9.7 (1.0) | 9.3 (0.7) |  |
|  | Mexican | 21.8 (1.7) | 33.4 (2.2) | 30.3 (2.2) | 36.1 (2.4) | 30.6 (1.7) |  |
|  | Puerto Rican | 26.0 (1.8) | 11.9 (1.0) | 20.3 (1.9) | 17.1 (1.5) | 18.7 (1.0) |  |
|  | South American | 4.9 (0.6) | 6.2 (0.6) | 4.2 (0.5) | 6.1 (0.6) | 5.5 (0.3) |  |
|  | Other | 1.9 (0.3) | 2.2 (0.4) | 2.3 (0.5) | 2.5 (0.8) | 2.3 (0.3) |  |
| **BMI (kg/m^2^)** | | 29.9 (6.8) | 29.7 (6.1) | 32.7 (7.0) | 28.2 (6.1) | 29.9 (6.7) | P<0.001 |
| **Cigarette Usage†** | |  |  |  |  |  |  |
|  | Never | 50.9 (1.6) | 54.9 (1.5) | 50.8 (1.7) | 57.4 (1.6) | 53.8 (0.9) | P<0.001 |
|  | Former | 25.0 (1.5) | 26.5 (1.4) | 31.2 (1.5) | 22.2 (1.3) | 25.7 (0.8) |  |
|  | Current | 24.1 (1.3) | 18.6 (1.2) | 18.0 (1.3) | 20.4 (1.2) | 20.5 (0.8) |  |
| **Alcohol Usage†** | |  |  |  |  |  |  |
|  | Doesn't drink alcohol | 58.5 (1.4) | 55.8 (1.5) | 55.0 (1.7) | 55.3 (1.9) | 56.2 (0.8) | p = 0.417 |
|  | Drinks alcohol | 41.5 (1.4) | 44.2 (1.5) | 45.0 (1.7) | 44.7 (1.9) | 43.8 (0.8) |  |
| **CVD event†** | |  |  |  |  |  |  |
|  | No CVD event | 45.5 (1.5) | 63.8 (1.3) | 51.5 (1.9) | 63.3 (1.7) | 56.6 (0.9) | P<0.001 |
|  | CVD event | 54.5 (1.5) | 36.2 (1.3) | 48.5 (1.9) | 36.7 (1.7) | 43.4 (0.9) |  |
| **Heart Failure†** | |  |  |  |  |  |  |
|  | No heart Failure | 96.2 (0.6) | 97.7 (0.5) | 96.1 (0.6) | 98.2 (0.3) | 97.1 (0.2) | p = 0.005 |
|  | Heart Failure | 3.8 (0.6) | 2.3 (0.5) | 3.9 (0.6) | 1.8 (0.3) | 2.9 (0.2) |  |
| **Stroke/TIA†** | |  |  |  |  |  |  |
|  | No Prevalent Stroke/TIA | 93.5 (0.7) | 95.5 (0.7) | 95.3 (0.7) | 97.8 (0.4) | 95.6 (0.3) | P<0.001 |
|  | Prevalent Stroke/TIA | 6.5 (0.7) | 4.5 (0.7) | 4.7 (0.7) | 2.2 (0.4) | 4.4 (0.3) |  |
| **Diabetes†** | |  |  |  |  |  |  |
|  | Non-diabetic | 71.1 (1.4) | 70.9 (1.4) | 61.0 (1.9) | 80.1 (1.1) | 71.8 (0.8) | P<0.001 |
|  | Diabetic | 28.9 (1.4) | 29.1 (1.4) | 39.0 (1.9) | 19.9 (1.1) | 28.2 (0.8) |  |
| **Hypertension†** | |  |  |  |  |  |  |
|  | Not hypertensive | 48.0 (1.5) | 51.0 (1.6) | 38.5 (1.7) | 64.2 (1.6) | 51.8 (0.9) | P<0.001 |
|  | Hypertensive | 52.0 (1.5) | 49.0 (1.6) | 61.5 (1.7) | 35.8 (1.6) | 48.2 (0.9) |  |
| **FRS** | | 0.2 (0.2) | 0.2 (0.2) | 0.2 (0.2) | 0.1 (0.1) | 0.2 (0.2) | P<0.001 |
| **FRS (3 categories)** | |  |  |  |  |  |  |
|  | **FRS < 0.1** | 42.3 (1.3) | 37.0 (1.4) | 26.9 (1.6) | 55.4 (1.5) | 41.9 (0.8) | P<0.001 |
|  | **0.1 <= FRS < 0.2** | 30.7 (1.3) | 31.7 (1.4) | 34.6 (1.8) | 26.0 (1.2) | 30.3 (0.7) |  |
|  | **FRS>=0.2** | 27.0 (1.3) | 31.4 (1.5) | 38.5 (1.8) | 18.6 (1.1) | 27.8 (0.8) |  |
| **HDL Cholesterol (mg/dL)*** | | 50.4 (17.4) | 48.8 (16.3) | 46.2 (13.0) | 51.7 (16.9) | 49.6 (16.3) | P<0.001 |
| **Total Cholesterol (mg/dL)*** | | 209.8 (56.5) | 211.2 (55.2) | 203.7 (51.3) | 208.8 (54.2) | 208.6 (54.5) | P<0.001 |
| **Triglycerides (mg/dL)*** | | 153.3 (236.6) | 155.3 (130.2) | 163.4 (134.4) | 140.8 (159.3) | 152.4 (172.4) | P<0.001 |
| **Education†** | |  |  |  |  |  |  |
|  | Less than HS | 43.0 (1.6) | 38.3 (1.5) | 40.6 (1.9) | 37.7 (1.6) | 39.8 (0.9) | p = 0.146 |
|  | HS or Equivalent | 20.9 (1.1) | 21.9 (1.2) | 19.5 (1.3) | 22.0 (1.5) | 21.2 (0.7) |  |
|  | Greater than HS | 36.1 (1.5) | 39.8 (1.6) | 39.9 (2.0) | 40.4 (1.5) | 39.0 (0.9) |  |
| **Income†** | |  |  |  |  |  |  |
|  | <$30,000 | 69.8 (1.7) | 62.8 (1.7) | 64.9 (1.8) | 61.9 (1.8) | 64.8 (1.2) | P<0.001 |
|  | >=$30,000 | 22.9 (1.6) | 30.1 (1.7) | 28.9 (1.6) | 32.7 (1.8) | 28.8 (1.2) |  |
|  | Not reported | 7.3 (0.9) | 7.1 (0.8) | 6.1 (1.0) | 5.4 (0.7) | 6.5 (0.4) |  |
| **Sash Language subscale*** | | 1.9 (1.3) | 1.6 (1.1) | 1.8 (1.2) | 1.8 (1.2) | 1.8 (1.2) | P<0.001 |
| **SASH Social subscale** | | 2.2 (0.8) | 2.1 (0.7) | 2.2 (0.7) | 2.2 (0.7) | 2.2 (0.7) | p = 0.008 |
| **Occupation Longest†** | |  |  |  |  |  |  |
|  | Non-skilled worker | 23.0 (1.4) | 22.9 (1.4) | 24.6 (1.4) | 24.7 (1.4) | 23.8 (0.8) | p = 0.223 |
|  | Service worker | 16.4 (1.1) | 15.8 (1.1) | 13.9 (1.2) | 15.1 (1.1) | 15.4 (0.6) |  |
|  | Skilled worker | 23.6 (1.5) | 21.5 (1.3) | 23.7 (1.7) | 21.6 (1.3) | 22.5 (0.8) |  |
|  | Professional/technical/other office worker | 17.2 (1.0) | 18.8 (1.3) | 14.6 (1.3) | 19.4 (1.3) | 17.8 (0.7) |  |
|  | Other | 19.8 (1.2) | 20.9 (1.2) | 23.1 (1.6) | 19.2 (1.7) | 20.5 (0.7) |  |
| **Occupation current + Employment status†** | | |  |  |  |  |  |
|  | Non-skilled worker | 7.8 (0.6) | 11.5 (0.8) | 10.3 (1.0) | 12.9 (0.8) | 10.7 (0.4) | P<0.001 |
|  | Service worker | 8.9 (0.9) | 9.6 (0.8) | 9.0 (1.0) | 11.8 (1.0) | 9.9 (0.5) |  |
|  | Skilled worker | 7.2 (0.6) | 11.1 (1.1) | 9.2 (1.0) | 9.7 (0.7) | 9.3 (0.4) |  |
|  | Professional/technical/other office worker | 4.3 (0.6) | 7.3 (0.8) | 3.4 (0.5) | 7.4 (1.0) | 5.8 (0.5) |  |
|  | Other | 5.8 (0.6) | 7.7 (0.7) | 7.6 (0.9) | 8.5 (1.4) | 7.4 (0.5) |  |
|  | Retired & Not employed | 21.1 (1.4) | 19.5 (1.2) | 27.3 (1.9) | 13.9 (1.0) | 19.7 (0.7) |  |
|  | Not retired & not employed | 45.1 (1.7) | 33.4 (1.5) | 33.2 (1.8) | 35.8 (1.6) | 37.1 (0.9) |  |

**Notes:**

* Means and Standard Deviations are presented **; †** % and Standard Errors (SEs) are presented

*P* value: Pearson's chi square test for continuous variables; Regression based F test for categorical variables

**HDL**: High-density lipoproteins; **BMI**: Body Mass Index; **CHD**: Coronary heart disease; **TIA**: Transient Ischemic Attack; **HS**: High School; **SASH**: Short Acculturation Scale for Hispanics
